# Advancements in Thermal Insulation through Ceramic Micro-Nanofiber Materials

**DOI:** 10.3390/molecules29102279

**Published:** 2024-05-12

**Authors:** Wenqiang Wang, Qiuxia Fu, Jianlong Ge, Sijun Xu, Qixia Liu, Junxiong Zhang, Haoru Shan

**Affiliations:** 1 School of Textile and Clothing, Nantong University, Nantong 226019, China; wwq1710@163.com (W.W.); fuqx@ntu.edu.cn (Q.F.); gejianlong@ntu.edu.cn (J.G.); xusijunwork@hotmail.com (S.X.); zhangjunxiong@ntu.edu.cn (J.Z.); 2 National and Local Joint Engineering Research Center of Technical Fiber Composites for Safety and Health, Nantong University, Nantong 226019, China

**Keywords:** ceramic micro-nanofibers, thermal insulation, structural design, construction strategies

## Abstract

Ceramic fibers have the advantages of high temperature resistance, light weight, favorable chemical stability and superior mechanical vibration resistance, which make them widely used in aerospace, energy, metallurgy, construction, personal protection and other thermal protection fields. Further refinement of the diameter of conventional ceramic fibers to microns or nanometers could further improve their thermal insulation performance and realize the transition from brittleness to flexibility. Processing traditional two-dimensional (2D) ceramic fiber membranes into three-dimensional (3D) ceramic fiber aerogels could further increase porosity, reduce bulk density, and reduce solid heat conduction, thereby improving thermal insulation performance and expanding application areas. Here, a comprehensive review of the newly emerging 2D ceramic micro-nanofiber membranes and 3D ceramic micro-nanofiber aerogels is demonstrated, starting from the presentation of the thermal insulation mechanism of ceramic fibers, followed by the summary of 2D ceramic micro-nanofiber membranes according to different types, and then the generalization of the construction strategies for 3D ceramic micro-nanofiber aerogels. Finally, the current challenges, possible solutions, and future prospects of ceramic micro-nanofiber materials are comprehensively discussed. We anticipate that this review could provide some valuable insights for the future development of ceramic micro-nanofiber materials for high temperature thermal insulation.

## 1. Introduction

Industrial production, firefighting and rescue operations, aerospace, and other fields are frequently confronted with high-temperature environments, which could pose health risks to individuals or lead to serious safety accidents. Therefore, various high-temperature insulation techniques and materials are used to protect personnel and equipment, ensuring the completion of work or emergency rescue tasks. High-temperature insulation materials are generally ceramic-based materials, which could be classified into three categories from the perspective of morphology, named porous, powdery, and fibrous ceramic materials. Porous ceramic insulation materials, including foam ceramics, honeycomb ceramics, and granular ceramics, are designed to have high porosity and small pore size, which enables them to exhibit favorable thermal insulation properties [1]. However, there exist some intrinsic drawbacks in porous ceramics, such as fragility, limited temperature range, and their being prone to structural collapse under wet conditions [2,3]. Powdered ceramic insulation materials are typically composed of brittle powders, such as glass beads, vermiculite powder, and perlite, that are processed and molded into hard materials. These materials are known for their lightweight nature, low thermal conductivity, and high temperature resistance. However, this type of material has high hygroscopicity and poor shock resistance [4,5,6]. Alternatively, fibrous ceramic insulation materials have been extensively applied in numerous fields benefitting from their low thermal conductivity, superior thermal stability, high thermal shock resistance, and favorable chemical resistance [7].

Ceramic fibers are typically produced by melting or spinning a mixture of ceramic raw materials (e.g., alumina, silica, or zirconia) to create long and thin fibers. At present, the common ceramic fiber insulation materials on the market mainly include basalt fiber, aluminum silicate fiber, high silica fiber, quartz fiber, mullite fiber, and so on. The traditional ceramic fibers are relatively coarse with diameter over 5 μm, brittle, and have high thermal conductivity, which greatly limits their practical applications in the field of thermal insulation. When the fiber diameter is further refined to the order of microns or nanometers, its thermal insulation performance could be significantly improved, and the mechanical properties of ceramic fibers could be effectively enhanced [8]. Therefore, research on ceramic micro-nano fiber thermal insulation materials is increasing. Various techniques have been developed for preparing ceramic micro-nano fibers, such as the template synthesis method, the vapor deposition method, sol-gel, and electrospinning [9]. Among these methods, electrospinning is considered to be an attractive and promising approach for the fabrication of ceramic nanofibers with tailored properties for various technological applications based on the significant advantages in the following aspects: relative simplicity, scalability, versatility in material selection, and controllable fiber structure [10].

Up to now, various types of ceramic micro-nano fibers have been developed, such as oxide ceramic fiber, nitride ceramic fiber, and carbide ceramic fiber. However, due to limitations in the existing manufacturing technology, the currently fabricated ceramic micro-nanofiber materials tend to be two-dimensional (2D) membranes, making it difficult to achieve larger thickness and higher porosity. Therefore, researchers are constantly striving to explore more effective manufacturing techniques to overcome this limitation and expand the application range of ceramic micro-nanofiber materials. Numerous approaches have been developed for preparing three-dimensional (3D) ceramic fiber-based aerogels, such as the direct spinning method, template method, gel drying method, freeze drying method, stacking method, and suction method.

**Figure 1:**
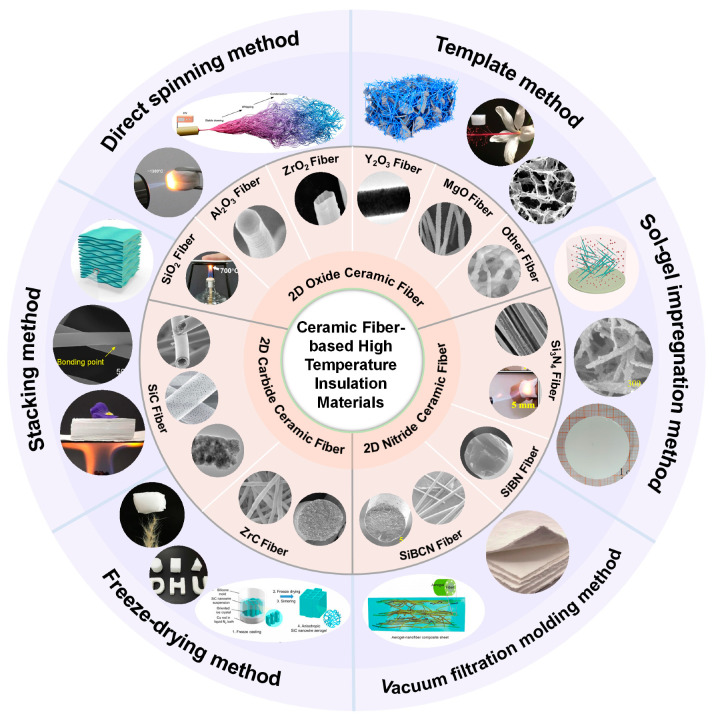
Ceramic fiber-based high temperature insulation materials, including 2D ceramic fibers (oxide ceramic fiber, carbide ceramic fiber, and nitride ceramic fiber) and 3D ceramic fibrous aerogels constructed via varieties of approaches. The images are of 2D ceramic fibers. Reproduced with permission [11]. Copyright 2022, Springer. Reproduced with permission [12]. Copyright 2012, WILEY-VCH. Reproduced with permission [13]. Copyright 2019, Elsevier. Reproduced with permission [14]. Copyright 2018, Elsevier. Reproduced with permission [15]. Copyright 2017, Elsevier. Reproduced with permission [16]. Copyright 2017, Springer. Reproduced with permission [17]. Copyright 2014, Elsevier. Reproduced with permission [18]. Copyright 2023, Elsevier. Reproduced with permission [19]. Copyright 2020, WILEY. Reproduced with permission [20]. Copyright 2021, Elsevier. Reproduced with permission [21]. Copyright 2023, WILEY. Reproduced with permission [22]. Copyright 2022, Elsevier. Reproduced with permission [23]. Copyright 2016, WILEY. Reproduced with permission [24]. Copyright 2017, Elsevier. Reproduced with permission [25]. Copyright 2014, The Royal Society of Chemistry (RSC). Reproduced with permission [26]. Copyright 2019, Elsevier. The images of 3D ceramic fibrous aerogels. Reproduced with permission [27]. Copyright 2022, Nature Portfolio. Reproduced with permission [28]. Copyright 2017, American Association for the Advancement of Science (AAAS). Reproduced with permission [29]. Copyright 2021, Elsevier. Reproduced with permission [30]. Copyright 2019, AAAS. Reproduced with permission [31]. Copyright 2021, American Chemical Society (ACS). Reproduced with permission [32]. Copyright 2020, ACS. Reproduced with permission [33]. Copyright 2020, AAAS. Reproduced with permission [34]. Copyright 2018, AAAS. Reproduced with permission [35]. Copyright 2022, Elsevier.

In this review, we systematically expounded on the thermal insulation mechanism of ceramic fibers to clarify the main ways to significantly improve the high-temperature thermal insulation performance of these materials. Subsequently, we thoroughly summarized the physicochemical properties, thermal insulation performance, and research status of the currently developed ceramic fibrous materials, including 2D ceramic fibers and 3D ceramic fibrous aerogels, as shown in Figure 1. And then, we provided a detailed summary of bulk ceramic fibrous materials, including the preparation strategy, and the advantages and disadvantages of the preparation methods. Finally, the present application status and the prospect of ceramic fibrous materials in high-temperature insulation are analyzed.

## 2. Heat Transfer in Ceramic Fiber Materials

Fiber-based insulation materials typically consist of two main components: the gas phase and the solid phase. The transfer of heat energy through these materials involves a combination of heat conduction, heat convection, heat radiation, and gas–solid coupled heat transfer. This combination of heat transfer mechanisms allows fiber insulation materials to effectively reduce the transfer of heat and improve thermal insulation in various applications.

### 2.1. Heat Conduction

**Figure 2:**
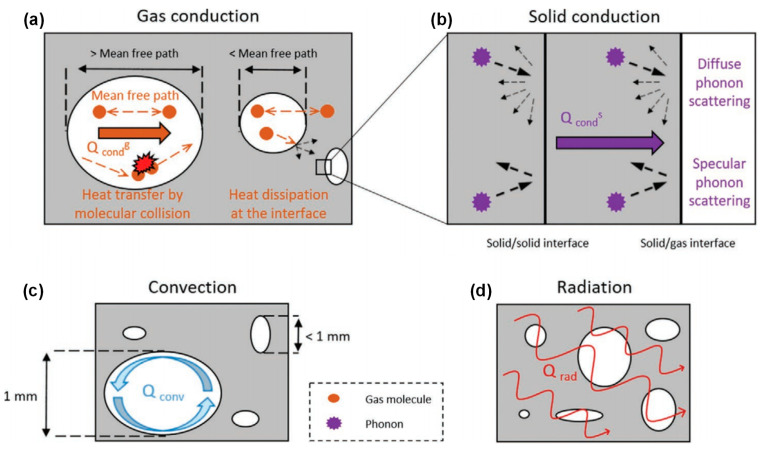
Heat transfer mode in porous materials: (**a**) gas heat transfer. (**b**) Solid heat conduction. (**c**) Heat convection. (**d**) Heat radiation. Reproduced with permission [39]. Copyright 2021, WILEY-VCH.

Heat conduction is the transfer of thermal energy through a material or between objects in direct contact when there is no relative displacement between their parts. It is caused by the unpredictable thermal movement of small particles, such as molecules, atoms, and free electrons. In a solid material, these particles vibrate and transfer energy to the neighboring ones, causing the heat to propagate through the material. In the absence of any macroscopic movement, the transfer of heat occurs purely through the conduction process [36]. Heat conduction could be categorized into gas heat conduction and solid heat conduction, based on the medium through which heat is transferred. For gas heat conduction, when there is a temperature gradient in a gas medium, the high-energy molecules transfer some of their kinetic energy to lower-energy molecules through collisions, as presented in Figure 2a. Similarly, when the gas comes into contact with a solid surface, heat transfer occurs through collisions between gas molecules and the solid surface. These collisions cause energy transfer, and contribute to the overall heat conduction in the gas phase. The thermal conductivity of gas could be mathematically represented by Equation (1) [8]:

(1) $$\lambda_{g}=\frac{\lambda_{g,0}}{\theta +2\psi \frac{\beta l_{g,0}f_{v}}{P_{r}\varepsilon d}}$$

where λ_g_ represents the thermal conductivity of gas and λ_g,0_ denotes the intrinsic thermal conductivity of individual gas molecules. The symbols θ, Ψ, and β correspond to parameters associated with the Couson number, while P_r_ represents the Prandtl number. The symbol ε represents a constant, f_v_ represents the fiber volume fraction, and d denotes the fiber diameter. l_g,0_ represents the average distance traveled by gas molecules between successive collisions. As indicated by Equation (1), the reduction of gas thermal conductivity can be achieved not only by substituting gas molecules with high thermal conductivity, but also by enhancing the mean free path of the gas, augmenting the fiber volume fraction, and decreasing the fiber diameter. Comparatively, the gas thermal conductivity can be significantly and conveniently reduced by elevating the fiber volume fraction and decreasing the fiber diameter. However, for ceramic fiber assemblies, a high fiber volume fraction could also increase the solid thermal conductivity between fibers. Hence, when engineering fiber thermal insulation materials, it is essential to strike an appropriate equilibrium between augmenting the fiber volume fraction and diminishing the fiber diameter in order to attain optimal thermal insulation performance.

Solid heat conduction is mainly achieved through phonon heat transfer, and the characteristics of phonon motion, including frequency, scattering mechanisms, and group velocity, determine the thermal conductivity of solids. Phonon scattering at lattice defects, grain boundaries, impurities, and other locations impacts their heat-carrying ability, as shown in Figure 2b. Scattering can alter the propagation direction and velocity of phonons, thereby affecting heat transfer and leading to a reduction in thermal conductivity. The thermal conductivity of a solid can be expressed by Equation (2) [8]:

(2) $$\lambda_{s}=\frac{1}{3}C_{S}V_{ph}l_{S}$$

where λ_s_ is the solid thermal conductivity, C_s_ is the constant volume specific heat capacity of the unit volume phonon, V_ph_ is the average velocity of the phonon, and l_s_ denotes the mean free path of the phonon. It can be seen from Equation (2) that decreasing the average phonon mean free path could efficiently reduce the thermal conductivity. As for the ceramic fiber insulation materials, increasing the complexity of the material’s crystal structure, defect concentration, impurity content, and the number of grain boundaries could decrease the average phonon mean free path, thereby improving the thermal insulation performance.

Given the extremely low electrical conductivity of conventional ceramics, the electronic contribution to thermal conductivity could generally be considered negligible [37]. However, in some cermet composites, graphite, 2D materials (e.g., graphene, transition metal dichalcogenides, black and blue phosphorus), metal carbides, semiconductor materials, and the contribution of electronic conduction to the effective thermal conductivity may be equal to or greater than that of lattice vibrations (phonons). The electrons responsible for heat and electricity conduction can be considered as a gas of nearly free electrons that move randomly within the periodic potential of the crystal lattice [37]. When exposed to electrical fields or temperature gradients, the electrons are driven in a specific direction, resulting in the transfer of charge or thermal energy. It is widely acknowledged that the electronic thermal conductivity (λ_e_) at a given absolute temperature (T) could be governed by the Wiedemann–Franz law [38]:

(3) $$L_{\left(T\right)}\equiv \frac{\lambda_{e}}{\sigma \cdot T}=\frac{\pi^{2}}{3}\cdot \frac{{K_{B}}^{2}}{e^{2}}\equiv L_{0}$$

where L_(T)_ represents the effective Lorentz number varying with T, σ is electrical conductivity, K_B_ is the Boltzmann constant, e is the electron charge, and L_0_ is the Sommerfeld value derived from Fermi liquid theory, and L_0_ = 2.4453 × 10^−8^ W·Ω·K^−2^.

### 2.2. Heat Convection

Thermal convection is the process of heat transfer through the movement of air or fluid, and can be divided into natural convection and forced convection [40]. Natural convection is the fluid motion caused by density differences in the flow field due to temperature changes, while forced convection is the convection caused by external factors [41]. When heat is applied to ceramic fibers, air or gas may form a convection loop between the fibers to transfer heat and maintain temperature uniformity. In the porous fiber assembly, the fluid and the fiber surface have thermal energy transfer to each other, as illustrated in Figure 2c.

Researchers have conducted in-depth research on the thermal convection process in fibrous materials. Fricke [42] proposed that when the bulk densities of porous-structured fiber aggregates exceed 2 × 10^4^ g·cm^−3^, the fibers can separate the gas fluid into small enough pores, and it can be considered that there is no heat convection inside the aggregate. This means that for the fibrous materials with high volumetric density, heat transfer is mainly achieved through thermal conduction, with a relatively small contribution from thermal convection. Therefore, in this case, the fiber material can be treated as a medium without internal thermal convection. However, when the volumetric density is low, the gaps between fibers are larger, allowing fluid to flow freely between the fibers and giving rise to thermal convection. This is due to density differences caused by temperature variations, leading to convective circulation of fluid within the fiber assembly, thereby facilitating heat transfer. Daryabeigi [43] also proposed that when the bulk density of the fiber insulation material is not less than 2.4 × 10^4^ g·cm^−3^, there is no natural convection inside the fiber material.

For the ceramic fibrous materials, adjusting the texture, length, and arrangement of fibers to create more voids and channels inside the assemblies could efficiently promote thermal convection effects, and thereby improve the thermal insulation performance. Moreover, the arrangement and compaction of ceramic fibers could be adjusted to enhance the connectivity and fluidity of convection channels, contributing to the increase of the strength and efficiency of the thermal convection effect. From the perspective of material surface modification, adding microstructures or surface coatings onto the surface of ceramic fibers might improve the process of convective heat transfer, further enhancing the thermal convection effect. Additionally, combining ceramic fibers with other thermal insulation materials, such as aerogels, porous materials, or metal layers, could form composite material structures to further optimize the thermal convection effect and improve the insulation performance.

### 2.3. Heat Radiation

Radiative heat transfer primarily depends on the material’s optical response to infrared radiation. Compared to the average free path of infrared radiation photons, fiber insulation materials have a relatively large optical thickness. Thermal radiation transmits energy by emitting electromagnetic waves from the surface of an object, as depicted in Figure 2d. In the process of thermal radiation, it is accompanied by the energy conversion between the internal energy of the object and the electromagnetic wave energy. The thermal radiation process is affected by the optical thickness, extinction coefficient, albedo, and photon scattering of materials [40]. At room temperature, the contribution of radiation heat transfer is generally much smaller compared to conduction and convection. However, as the temperature increases, the significance of radiation heat transfer becomes more prominent. At high temperatures, the energy emitted by an object in the form of electromagnetic waves increases significantly. This is described by the Stefan–Boltzmann law, which states that the amount of heat radiated by an object is proportional to the fourth power of its temperature [44]. As a result, the rate of heat transfer by radiation increases with temperature. The radiation thermal conductivity (λ_r_) can be expressed by Equation (4) [8]:

(4) $$\lambda_{r}=\frac{16K_{B}n^{2}T^{3}}{3\rho e}$$

where K_B_ is the Stefan–Boltzmann constant, n represents the refractive index of the material, T denotes temperature, ρ stands for density of the material, and e is extinction coefficient. Through increasing the refractive index or decreasing the extinction coefficient of the ceramic fibrous materials could efficiently reduce the radiation thermal conductivity.

Constructing high reflectivity coatings or using coatings with infrared energy absorption capabilities can effectively reduce the fiber’s infrared transmittance and enhance its ability to reflect infrared radiation [45,46]. Currently, there are several types of high reflectivity coatings that have been constructed on the fiber surface, including TiO_2_, In_2_O_3_, CeO_2_, and SiC. It should be noted that the constructed high reflectivity coatings on the fiber surface has increased the ability to reflect infrared radiation, while the high thermal conductivity of coatings would enhance the solid-state heat conduction between the fibers, resulting in the coated fiber having a higher thermal conductivity compared to the fiber without the coating. It suggests that, in some cases, the choice of coating material needs to balance the desired infrared reflectance with the overall thermal conductivity requirements of the fiber materials.

### 2.4. Thermal Conductivity Modeling of Ceramic Fibrous Materials

Heat transfer in fiber insulation materials involves solid conduction through fibers, gaseous conduction, natural convection in the spaces between fibers, and radiative heat exchange in the participating media. The effective thermal conductivity (λ_eff_) of the fibrous material is given by

(5) $$\lambda_{\mathrm{eff}}=\lambda_{c}+\lambda_{convection}+\lambda_{r}$$

where λ_c_ is effective thermal conductivity due to solid and gaseous conduction, λ_convection_ is effective thermal conductivity due to convection, and λ_r_ is effective thermal conductivity due to radiation. As previously documented in the literature, the natural convection phenomenon is deemed to be insignificant in fibrous insulation materials with a density exceeding 20 kg m^−3^ [43]. This is attributed to the effective partitioning of gas molecules into sufficiently small interstices by the fibers. Thus, the effective thermal conductivity of the fibrous material could be expressed as follows:

(6) $$\lambda_{eff}=\lambda_{c}+\lambda_{r}$$

The structure of fibrous materials is complex, and they are commonly thought of as simplified semi-transparent media consisting of elongated, slender fibers, typically a few micrometers in diameter, interspersed with air or other gases that fill the interstitial space between the fibers. To facilitate the study of the thermal conductivity of fiber insulation materials, several basic theoretical thermal conductivity models due to solid and gas conductions for fibrous media have been developed, such as the Parallel model [43,47,48,49,50,51,52], Series model [47,53,54,55], Singh model [56,57], Bankvall model [54,58,59,60], Maxwell–Eucken model [55], Effective medium theory (EMT) model [51,61,62], Hamilton model [53,61,63], and Bhattacharyya model [43,64,65].

As summarized in Table 1, the Parallel model assumes that the fibers in the insulation material are parallel to each other, and accounts for the heat transfer through the fibers and the surrounding air or gas. The Series model considers the fibers in the insulation material to be in series and that heat sequentially flows through each fiber. This model accounts for the thermal resistance of each individual fiber and the cumulative effect on overall insulation performance. In many cases, there may be a certain angle (θ) between the fiber arrangement direction and the heat flow direction. Therefore, Jagjiwanram and Singh [56] proposed a new model based on the Parallel model and the Series model. Moreover, Bankvall et al. [58] postulated that the conduction of solids and gases is both parallel and series, where α is the ratio of parallel heat conduction. The Maxwell model assumes that the filler is randomly dispersed in the matrix, and then calculates the thermal conductivity of the composite without considering the interaction of the dispersed particles in the matrix [55]. The EMT model assumes that the two-phase components can be distributed continuously or discontinuously in the composite, which can be transmitted independently or dispersed in a material [51,61]. Hamilton et al. [63] introduced the empirical shape factor (n) into the model. For spherical particles, n = 3; for infinitely long cylinders, n = 6. Bhattacharyya et al. [64] considered the fiber orientation and introduced the orientation parameter into the model. When the fibers are randomly oriented, the orientation parameter is considered to be 2/3. The aforementioned models permit researchers to describe the effective thermal conductivity of fiber materials in accordance with the specific structural parameters of fiber insulation materials, and to introduce appropriate structural correction factors.

## 3. 2D Ceramic Fiber Membranes

**Figure 3:**
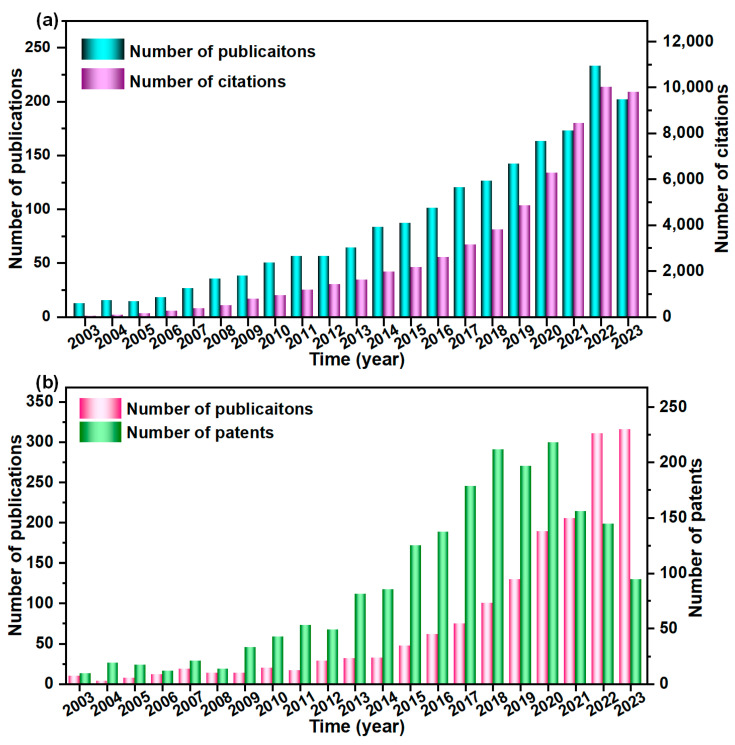
(**a**) The count of search results of publication and citations related to ‘ceramic micro-nanofibers’ on Web of Science from year 2003 to 2023. (**b**) The count of search results of publication and patents related to ‘ceramic fiber’ and ‘thermal insulation’ on web of science and incoPat from year 2003 to 2023.

When further decreasing the fiber diameter, the number of interfibrous contact points is decreased, resulting in an increase in the heat transfer path and the encountered interface barrier. Moreover, thinner fibers with increased surface areas could enhance their ability to absorb and scatter incoming infrared radiation, thus reducing the infrared transmittance. Therefore, preparing fibers with a diameter at the micron or even nanometer level is one of the effective means to enhance the thermal insulation capabilities of fibrous materials. Recently, with the continuous innovation of preparation methods, ceramic micro-nano fiber-based insulation materials have been developed rapidly (Figure 3). The currently developed ceramic micro-nano fibers could be roughly divided into two categories, including oxide ceramic fibers and non-oxide ceramic fibers. In this section, we present a comprehensive review on the categorization of ceramic fibers into oxide ceramic fibers, carbide ceramic fibers, and nitride ceramic fibers.

### 3.1. Oxide Ceramic Fiber Insulation Material

The oxide ceramic fibers reported in the current literature for high-temperature insulation mainly include silicon dioxide (SiO_2_), aluminum oxide (Al_2_O_3_), zirconium oxide (ZrO_2_), yttrium oxide (Y_2_O_3_), and magnesium oxide (MgO). These have been extensively studied for their exceptional thermal properties and suitability for various industrial applications requiring elevated temperature environments.

#### 3.1.1. SiO_2_ Fiber

**Figure 4:**
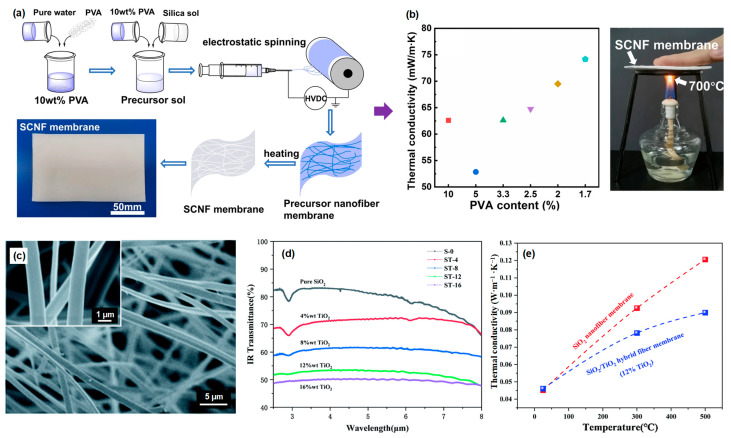
(**a**) Schematic depicting the fabrication procedure for SiO_2_ nanofibrous membranes. (**b**) Variation in thermal conductivity among SiO_2_ nanofibrous membranes prepared using different precursor sols and emphasizing the excellent thermal insulation properties exhibited by SiO_2_ fiber membranes. (**c**) SEM images of SiO_2_/TiO_2_ fiber membrane at 900 °C. (**d**) Infrared transmittance spectra of specimens with varying TiO_2_ contents. (**e**) Thermal conductivity profiles of the dual fiber membranes at different temperature intervals. (**a**,**b**) Reproduced with permission [11]. Copyright 2022, Springer. (**c**–**e**) Reproduced with permission [66]. Copyright 2021, RSC.

SiO_2_ fibers exhibit advantageous characteristics, such as remarkable thermal stability at elevated temperatures, limited heat transfer properties, significant surface area, and corrosion resistance, which enables their considerable potential in high-temperature insulation applications, particularly within industries such as aerospace, chemical, and energy sectors. Among numerous preparation methods, the sol-gel electrospinning method is widely considered a highly efficient technique for the fabrication of SiO_2_ nanofibers. Yu and co-workers [11] prepared an ultra-soft SiO_2_ nanofiber membrane by using polyvinyl alcohol (PVA) as template and commercial SiO_2_ sol as a silicon source (Figure 4a). Among the various fabricated SiO_2_ fibrous membranes, the sample prepared using a 5% PVA precursor sol and sintering temperature of 900 °C exhibits a tension strength of 4.145 MPa and a thermal conductivity of 0.05285 W·m^−1^·K^−1^ (Figure 4b). Due to the fact that pure SiO_2_ exhibits high transparency to near-infrared (NIR) radiation within a wavelength range of 1 to 8 mm, enhancing the anti-infrared radiation properties of SiO_2_ nanofiber membranes is crucial for expanding their potential applications and improving their high-temperature insulation performance. To address this issue, Song and co-workers [66] employed an effective approach, in which they combined SiO_2_ sol and TiO_2_ sol to attain a uniform dispersion of the infrared opacifier TiO_2_ within the SiO_2_ nanofiber matrix (Figure 4c). The findings from their study demonstrate a clear relationship between the TiO_2_ content and the infrared transmittance of the SiO_2_/TiO_2_ fibrous membranes. With the gradual increase in TiO_2_ concentration, a notable decrease in infrared transmittance was observed (Figure 4d), highlighting the significant impact of TiO_2_ addition on reducing the passage of infrared radiation through the fiber membrane. The resultant SiO_2_/TiO_2_ fibrous membranes possess a thermal conductivity of 0.045 W·m^−1^·K^−1^ at room temperature (Figure 4e). More notably, when compared to pure SiO_2_ fibrous membranes, the introduction of TiO_2_ resulted in a notable reduction in thermal conductivity at a temperature of 500 °C. Specifically, for the composite fibers with a TiO_2_ content of 12%, the thermal conductivity decreased by 25%, ranging from 0.12 W·m^−1^·K^−1^ to 0.089 W·m^−1^·K^−1^. Additionally, to improve the tensile strength of SiO_2_ fibrous membranes, Si and co-workers [67] added NaCl into the SiO_2_ sol to form a bonded structure between SiO_2_ nanofibers for effectively reducing the slippage of fibers during the tensile process, thereby increasing the tensile strength of the nanofiber membrane to 5.5 MPa. Furthermore, the fabricated membranes exhibited outstanding flexibility and insulation performance.

#### 3.1.2. Al_2_O_3_ Fiber

**Figure 5:**
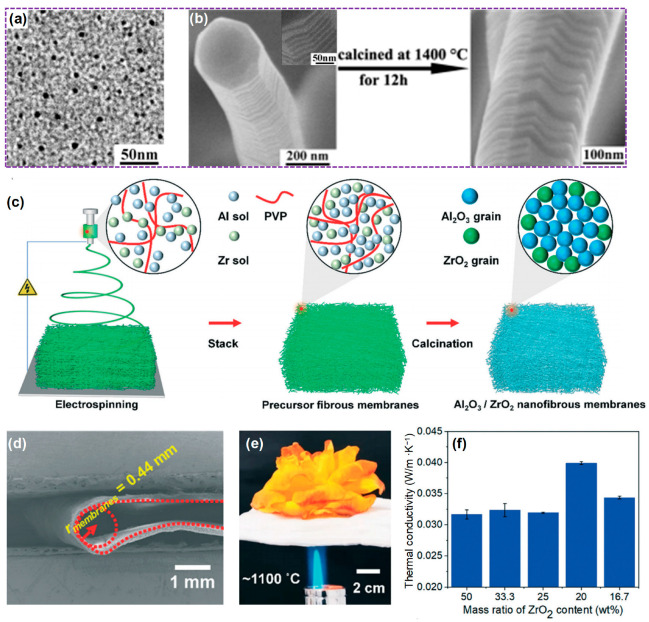
(**a**) TEM image of the sol particles (black dots) in the electrospinning precursor. (**b**) SEM images of α-Al_2_O_3_ nanofibers before and after calcining at 1400 °C for 12 h, demonstrating their extraordinary thermal stability. (**c**) Schematic depicting the preparation procedure of Al_2_O_3_/ZrO_2_ nanofibrous membranes. (**d**) SEM images of Al_2_O_3_/ZrO_2_ membrane bending and folding at the microscale. (**e**) Illustration of the outstanding thermal insulation performance of the Al_2_O_3_/ZrO_2_ fibrous membrane. (**f**) Thermal conductivity of Al_2_O_3_/ZrO_2_ nanofibrous membranes. (**a**,**b**) Reproduced with permission [12]. Copyright 2012, WILEY-VCH. (**c**–**f**) Reproduced with permission [70]. Copyright 2022, RSC.

In high-temperature settings, Al_2_O_3_ fibers demonstrate pronounced benefits in oxidation resistance, specific heat capacity, thermal conductivity, and other properties, thereby garnering considerable interest from researchers. According to previous research [68], Al_2_O_3_ exhibits diverse crystal structures, including η, γ, χ, δ, κ, θ and α, among which only the α-Al_2_O_3_ phase is thermodynamically stable, while the others are metastable. With rising temperatures, the metastable state will transition to a stable state; however, the transition temperature for stable α-Al_2_O_3_ is exceedingly high, which typically surpasses 1200 °C, poses challenges to the fabrication of fine fibers. As a result, achieving the desired characteristics of Al_2_O_3_ fibers in terms of size, morphology, and uniformity becomes increasingly difficult. Mitigating the transition temperature to a more manageable range is crucial for enhancing the feasibility and efficiency of the fiber preparation processes. Zhang and co-workers [12] fabricated α-Al_2_O_3_ nanofibers composed of nanosheets by the integration of the sol-gel approach and the electrostatic spinning technique (Figure 5a). To impede the conversion of γ-Al_2_O_3_ to α-Al_2_O_3_, the addition of magnesium sulfate (MgSO_4_) was employed as a stabilizing agent. Consequently, the formation of MgAl_2_O_4_ at the grain boundaries facilitated the inhibition of grain growth and effectively enhanced the sintering process. The resulting α-Al_2_O_3_ nanofibers exhibit superior thermal stability, retaining their microstructure even after subjecting them to a prolonged heat treatment of 1400 °C for 12 h (Figure 5b). Afterwards, Wang and co-workers [69] employed aluminum isopropoxide as the precursor to fabricate Al_2_O_3_ nanofibers through electrospinning and heat treatment processes. The resulting fibers exhibited a thermal conductivity of 0.318 W·m^−1^·K^−1^ at 1000 °C, and the α-Al_2_O_3_ transformation temperature is as low as 1000 °C, which is 100 to 200 °C lower than the conventional transformation temperature.

Unlike conventional aluminum sources that rely on metal aluminum salts, Li and co-workers [71] select pseudo-boehmite nanoparticles as the aluminum source to fabricate Al_2_O_3_-based nanofibers. Nitric acid was chosen as the peptizing agent and PVP as the spinning additives to prepare the spinnable solution. After the electrospinning process and the subsequent calcination at 1300 °C, continuous Al_2_O_3_-SiO_2_ composite nanofibers was obtained. The thermal conductivity of the Al_2_O_3_-SiO_2_ fibrous composite calcined at 1300 °C is 0.08867 W·m^−1^·K^−1^. Recently, Li and co-workers [70] prepared Al_2_O_3_/ZrO_2_ fibrous membrane through sol-gel electrospinning approach (Figure 5c). The resulting membranes display favorable flexibility, dramatic fire and temperature resistance, as well as low thermal conductivity of 0.03166 W·m^−1^·K^−1^ at room temperature (Figure 5d–f).

#### 3.1.3. ZrO_2_ Fiber

ZrO_2_ fibers possess the integrated characteristics of low thermal conductivity, good insulation properties, a high melting point, a high refractive index, excellent thermal shock resistance, and stable chemical properties, making them highly desirable in industries such as aerospace, automotive, and defense. ZrO_2_ exists in three distinct crystal structures, namely monoclinic, tetragonal, and cubic. It is notable that the transformation from the tetragonal phase to the monoclinic phase results in a volumetric expansion, leading to structural deterioration in ZrO_2_. To mitigate the detrimental effects of the tetragonal-to-monoclinic phase transformation in ZrO_2_, the addition of specific crystal stabilizers is commonly employed. These stabilizers include yttrium oxide, calcium oxide, and magnesium oxide. Their incorporation during the fabrication process of ZrO_2_ helps prevent the occurrence of this undesired phase transition and subsequent structural integrity degradation [72].

**Figure 6:**
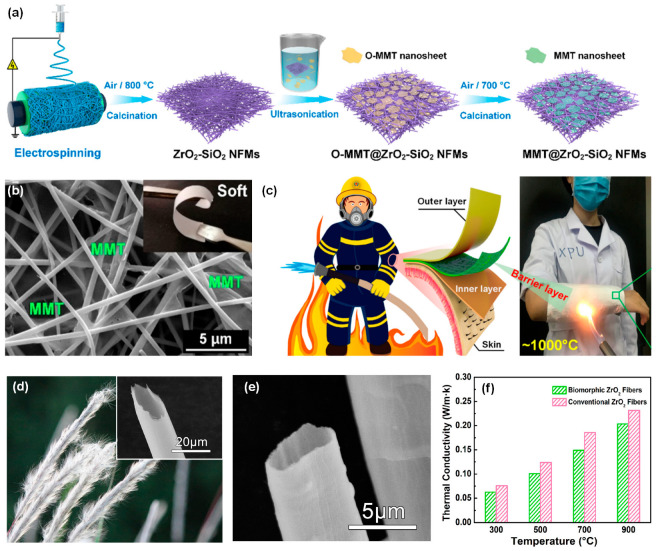
(**a**) The synthetic steps for MMT@ZrO_2_−SiO_2_ fibrous membranes. (**b**) SEM image and flexible display photograph of the resultant fibrous membrane. (**c**) Presentation of MMT@ZrO_2_-SiO_2_ fibrous membranes in firefighter uniforms and its thermal insulation performance in a 1000 °C flame. (**d**) Optical image of cogon (the inset depicts a high-magnification SEM image of cogon fiber). (**e**) high-magnification SEM image and (**f**) thermal conductivities of the biomorphic ZrO_2_ fibers. (**a**–**c**) Reproduced with permission [73]. Copyright 2021, ACS. (**d**–**f**) Reproduced with permission [13]. Copyright 2019, Elsevier.

Mao and co-workers [73] utilize electrospinning to fabricate flexible yttrium-stabilized zirconia (YSZ) nanofibers and achieved a transition from brittle to flexible to brittle by controlling the yttrium content in the fibers. The brittle-to-flexible-to-brittle transition of YSZ membranes is studied with regard to the influences of crystalline phase, grain size, and pore width. They found that the optimal grain size for the YSZ nanofibrous membranes is 23.8 nm. Moreover, to further enhance the mechanical and insulation properties of ZrO_2_-SiO_2_ nanofibers, they incorporate the ultrathin montmorillonite (MMT) nanosheets into ZrO_2_-SiO_2_ fiber substrate and establishing interfaces with fibers (Figure 6a). The synthesized MMT@ZrO_2_-SiO_2_ membranes exhibited favorable softness, tension strength of 1.83 MPa, exceptional fire resistance, and remarkable thermal insulation capabilities (~0.026 W·m^−1^·K^−1^ at room temperature), as demonstrated in Figure 6b,c. Subsequently, following a direct polymer template-regulated sol-gel electrospinning process and follow-up calcination, a range of yttria-doped zirconia (YDZ) nanofibrous membranes were successfully fabricated, demonstrating flexibility, foldability, and exceptional thermal stability [74]. The study illustrated the controllability of fibrous dimensions, grain morphology, mechanical characteristics, and thermal resistance of YDZ fibrous membranes through manipulation of polymer template composition and molecular weight. The resultant YDZ fibrous membranes demonstrate extraordinary performance, including outstanding tensile strength of approximately 4.82 MPa, excellent thermal durability up to a temperature of 1200 °C, low thermal conductivity ranging from 0.008 to 0.023 W·m^−1^·K^−1^ across the temperature range of 25 to 1000 °C, and outstanding fire resistance.

Peng [75] incorporated a high concentration of amorphous silica to impede grain growth, leading to the fabrication of a YSZ/SiO_2_ nanofiber membrane. This ceramic fiber membrane exhibits a degree of flexibility, with a measured tensile strength of 4.0 ± 0.7 MPa. Additionally, the membrane demonstrates a thermal conductivity ranging from 0.0287 to 0.0469 W·m^−1^·K^−1^. In a separate study, Peng [76] successfully fabricated a two-phase hydrophobic SiO_2_-ZrO_2_ composite ceramic nanofiber membrane. The fiber membrane exhibits a considerable strength ranging from 5 to 8.4 MPa, along with a thermal conductivity of 0.030 W·m^−1^·K^−1^. Notably, it possesses a remarkable heat radiation reflectivity of 90% and demonstrates high hydrophobic temperature resistance up to 450 °C. Even in a water vapor environment of 350 °C, the membrane maintains its complete hydrophobicity. These exceptional properties make it a promising material for the production of fire-fighting thermal insulation clothing.

Additionally, some scholars have enhanced the thermal insulation performance of ZrO_2_ fibers by constructing a hollow structure within them. Wang et al. [13] successfully synthesized a novel variety of hollow ZrO_2_ fiber characterized by exceptional thermal resistance and stability through a template approach. They employed cogon fiber as a natural template, resulting in the formation of ZrO_2_ fibers with inherited hollow structures (Figure 6d,e). The hollow structure inherent in the biomorphic ZrO_2_ fiber significantly enhances its ability to impede heat flow when compared to solid fibers, thereby leading to a noteworthy reduction in thermal conductivity, as shown in Figure 6f. Consequently, these fibers demonstrate superior heat-insulating properties compared to traditional solid fibers.

#### 3.1.4. Y_2_O_3_ Fiber

**Figure 7:**
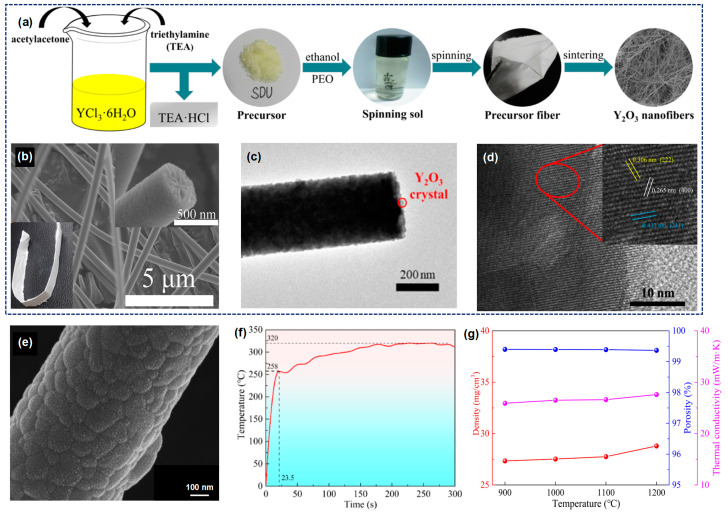
(**a**) Scheme illustrating the preparation of Y_2_O_3_ nanofibers. (**b**) SEM images of Y_2_O_3_ nanofibers treated at 800 °C for 2 h (the inset is the fiber cross section and optical photo). (**c**,**d**) TEM images of Y_2_O_3_ nanofibers heat treated at 800 °C. (**e**) SEM image of YAG fiber after heat treatment at 1200 °C. (**f**) Relationship between temperature change of cold surface and time under a butane torch. (**g**) The thermal conductivity, density, and porosity of YAG fiber membranes calcinated at different temperature. (**a**–**d**) Reproduced with permission [14]. Copyright 2018, Elsevier. (**e**–**g**) Reproduced with permission [77]. Copyright 2021, Elsevier.

Y_2_O_3_ fiber, as a significant rare earth oxide, exhibits traits such as a high melting point, robust thermal corrosion resistance, exceptional high temperature stability, and a high dielectric constant. These properties render it widely applicable in high-temperature heat insulation, flame retardant materials, and various related fields. Furthermore, the nanoscale Y_2_O_3_ demonstrates high reflectivity within the NIR region, indicating its potential utility as a material with NIR-reflective properties to mitigate the failure of metallic foils in high temperature oxidation environments [14]. Therefore, Xie and co-workers [14] fabricated high near-infrared reflective Y_2_O_3_ nanofiber membranes through electrospinning and heat treatment processes. As depicted in Figure 7a, a polyacetylacetone-yttrium precursor was synthesized and used to prepare a spinnable solution. The fabricated Y_2_O_3_ nanofibrous membranes exhibit extraordinary flexibility, which could be folded into various shapes with ease (Figure 7b). After calcination, the Y_2_O_3_ nanocrystals are clearly visible on the surface and cross-section of the fibers (Figure 7c,d). The wavelength-dependent reflectance of a Y_2_O_3_ nanofiber membrane spanning the range of 300 to 2500 nm are examined. Notably, upon undergoing calcination at 800 °C, the Y_2_O_3_ nanocrystals exhibited exceptional optical characteristics on both the surface and cross-section of the fibers. In the near-infrared (NIR) region, specifically within the wavelength range of 750–2500 nm, the treated membrane showcased an average reflectivity surpassing 92% and a maximum reflectivity peaking at 95%. These remarkable findings highlight the impressive near-infrared reflective effect achieved by Y_2_O_3_ nanofibrous membranes. By incorporating a 100 μm thick Y_2_O_3_ nanofiber membrane onto a zirconia fiber sheet with a hot surface temperature of 1500 °C, it becomes feasible to lower the temperature of the cold surface by approximately 20 °C.

Furthermore, they synthesized the metal-acetylacetone coordination polymerization precursor and fabricated yttrium aluminum garnet (YAG) nanofibrous membranes through electrospinning technique and the subsequent heat treatment (Figure 7e) [77]. During the calcination process, almost no mesophase is formed in YAG fibers. The resultant YAG nanofibrous membranes display a remarkable tensile strength measuring 2.43 MPa and demonstrate excellent heat resistance and thermal stability, which could effectively maintain a constant temperature of 320 °C on the cold surface when exposed to a hot surface temperature of 1200 °C (Figure 7f). The thermal conductivities of the YAG fibrous membrane remained stable within the range of 0.0252–0.0276 W·m^−1^·K^−1^ after undergoing a 2 h heat treatment at temperatures ranging from 900 to 1200 °C (Figure 7g). This level of conductivity is comparable to that observed in other high-performance materials such as SiO_2_ nanowire aerogel, Al_2_O_3_ aerogel, and ceramic fiber aerogel.

#### 3.1.5. MgO Fiber

Due to their elevated melting point, exceptional thermal stability, and resistance to oxidation and thermal degradation, MgO fibers serve as reinforcing agents in high-temperature insulating refractory materials and high-strength composite materials. Shao et al. [78] employed magnesium acetate as the metal precursor and polyvinyl alcohol (PVA) as the spinning agent to fabricate a spinnable solution. Subsequent to the electrospinning procedure and calcination performed at 800 °C, MgO nanofibers with diameters ranging from 50 to 200 nm were successfully synthesized. Afterwards, Zheng et al. [79] fabricated MgO fibers utilizing the synthesized magnesium propionate as the precursor, followed by pretreatment of the as-spun precursor fibers within two distinct settings of water vapor and atmospheric air. In the presence of water steam, it was anticipated that the propionate groups within the precursor fibers would be eliminated as molecules, yielding MgO fibers with a sleek and compact surface. Conversely, MgO fibers treated in air exhibit a notable presence of internal pores resulting from the release of gases during the combustion decomposition of organic groups. It is observed that the thermal conductivity of fibers treated in air is lower compared to those treated with water steam.

Subsequently, Xu et al. [15] employ two polymeric magnesium salts, magnesium acetate (MA) and magnesium citrate (MC), as precursor materials to effectively synthesize polycrystalline MgO nanofibers via the electrospinning method and heat treatment. The compactness of MgO nanofibers exhibited variation in response to alterations in the mass ratio of magnesium acetate and chitosan within the precursor solution. MgO nanofibers obtained from a magnesium acetate (MA) to magnesium citrate (MC) mass ratio of 1:2 demonstrated enhanced compactness and superior morphological characteristics following heat treatment at 1000 °C. MgO nanofibers featuring a mass ratio of 1:1 demonstrate the highest surface area and the lowest thermal conductivity among the tested specimens.

#### 3.1.6. Other Oxide Ceramic Fibers

In addition to the aforementioned oxide ceramic fibers, researchers have pioneered the development of a variety of composite oxide fibers, aiming to advance fiber mechanical properties, high-temperature insulation performance, and other application capabilities, such as mullite fibers [16], La_2_Zr_2_O_7_ fibers [80], Y_2_Zr_2_O_7_ fibers [81], Gd_2_Zr_2_O_7_/ZrO_2_ fibers [82], YAG-Al_2_O_3_ fibers [83], YSZ-Al_2_TiO_5_ fibers [84], etc.

Continuous mullite (3Al_2_O_3_·2SiO_2_) fibers play a significant role in ceramic materials due to their exceptional dielectric properties, low thermal conductivity, minimal thermal expansion coefficient, remarkable chemical stability, and superior thermal resistance at elevated temperatures. Thus, the research on mullite fibers has garnered increasing attention from academia and industry. Typically, utilizing the electrospinning technique with a nonhydrolytic sol precursor, Wei et al. [16] successfully fabricated mullite nanofibers characterized by superior flexibility and exceptional high-temperature resistance, demonstrating promising prospects for applications in advanced materials and thermal management systems. The electrospun mullite nanofibers exhibit a small crystal grain size, uniform diameter distribution, and limited presence of the amorphous phase, contributing to their exceptional flexibility. More importantly, following calcination at 1500 °C, the fabricated mullite nanofibers retain their fibrous morphology, thereby demonstrating potential for applications in high-temperature industrial and aerospace sectors. Subsequently, Wang et al. [85] have examined the influence of polymer template variations on the microscopic morphology and mechanical characteristics of mullite nanofibers. PVP and polyvinyl butyral (PVB) were chosen as templates for the production of mullite-based precursor fibers in this study. The results demonstrate that after the calcination process at 1000 °C, fibers incorporating PVB manifest a porous architecture favoring a higher surface area and mullite crystal formation, while those incorporating PVP exhibit denser features with the presence of both γ-Al_2_O_3_ and mullite crystals. These observations underscore the significance of template selection in tailoring the properties of mullite-based fibers for advanced applications. The differences in phase composition, specific surface area, and mechanical properties induced by polymer templating are believed to stem from the distinct coordination modes of polymer and Al-Si sol, leading to varied distributions of components within the spun fibers.

Yuan et al. [80] employ three different lanthanide salts (LaCl_3_·7H_2_O, La(NO_3_)_3_·7H_2_O, and La(CH_3_COO)_3_·4H_2_O) as metal sources to formulate precursor spinning solutions, which were subsequently processed into nanofibers using electrospinning technology. The results indicate that the La_2_Zr_2_O_7_ fibers prepared using LaCl_3_·7H_2_O as precursor possess finer grain sizes as compared to the other two metal sources, resulting in a relatively higher mechanical strength of the fibers. However, La_2_Zr_2_O_7_ fibers derived from La(CH_3_COO)_3_·4H_2_O precursor exhibit the lowest thermal conductivity, possibly attributed to the larger pore size and porosity on the fibers. Xie et al. [81] proposed an effective approach to fabricate flexible Y_2_Zr_2_O_7_ fibers using the synthesized polyacetylacetone yttrium-zirconium as precursor. The resulting Y_2_Zr_2_O_7_ fibrous membranes demonstrate exceptional thermal stability, retaining their phase integrity up to 1200 °C without undergoing any phase transition. Furthermore, the Y_2_Zr_2_O_7_ fibrous membranes exhibit remarkable heat radiation reflectivity with an average diffuse reflectance of 96.34% in the NIR band ranging from 750 to 2500 nm. The Y_2_Zr_2_O_7_ fibrous membranes integrate the exceptional thermal insulation properties (thermal conductivity of 0.038 W·m^−1^·K^−1^) arising from its ultra-low density and high resistance to elevated temperatures characteristic of ceramics.

### 3.2. Nitride Ceramic Fiber Insulation Material

Nitride ceramic fibers exhibit outstanding characteristics such as excellent high-temperature stability, high strength, and lightweight properties, making them extensively utilized in applications including aircraft engines and aerospace thermal protection structures. They provide reliable high-temperature insulation protection, ensuring the stable operation of equipment in extreme temperature environments. The currently developed ceramic nitride fibers mainly include silicon nitride (Si_3_N_4_) fibers, silicon boron nitride (SiBN) fibers, SiBCN fibers, boron nitride (BN) fibers, etc. Nitride ceramic fibers are predominantly prepared via the precursor conversion method, where specialized organic polymer fibers with unique composition structures are initially spun and formed into polymer fibers, followed by thermal treatment and inorganic conversion processes to obtain ceramic fibers. Typically, nitrogen-containing polymer precursors are employed as raw materials or nitrogen elements are introduced through the nitridation reaction of polymer fibers.

#### 3.2.1. Si_3_N_4_ Fiber

Silicon nitride fibers, known for their excellent mechanical strength, thermal stability, and corrosion resistance, are extensively utilized in the aerospace industry for manufacturing lightweight components with superior performance under harsh operating conditions.

Various methodologies have been employed for the synthesis of Si_3_N_4_ nanowires, including nitrogenation of nanocrystalline Si powder produced by cryomilling, thermal decomposition of polymeric precursor, thermal decomposition-nitriding of silicon monoxide (SiO), chemical vapor deposition, combustion synthesis, carbothermal reduction of SiO_2_-containing compound with carbon sources, utilization of rice husk ash or carbonaceous SiO_2_ xerogel in nitrogen-rich environments, as well as high-temperature treatment of a Si wafer in NH_3_ and N_2_ atmospheres [17].

Wang et al. [86] demonstrate an economical and scalable synthesis method for α-Si_3_N_4_ nanofibers and nanobelts, utilizing mesoporous silica-carbon nanocomposites as precursor materials through a carbothermal reduction and nitridation process. At elevated temperatures, the reaction between SiO_2_ and C initiates the formation of SiO and CO gases, followed by the subsequent reaction of SiO gas with N_2_ in the presence of C, leading to the nucleation of Si_3_N_4_ crystallites. Additionally, SiO_2_ xerogel and SiO_2_ nanoparticles are employed as precursor SiO_2_ sources to synthesize Si_3_N_4_ nanofibers through the carbothermal reduction process. Li and co-workers [17] have proposed a novel vapor-phase synthesis method for producing millimeter-scale single-crystalline α-Si_3_N_4_ nanowires. This process involves heating pre-oxidized silicon powders in a corundum crucible at 1390 °C under a nitrogen atmosphere, rather than using ammonia or a reducing atmosphere such as methane or hydrogen. It also excludes the inclusion of carbon or any metal catalysts in the process.

**Figure 8:**
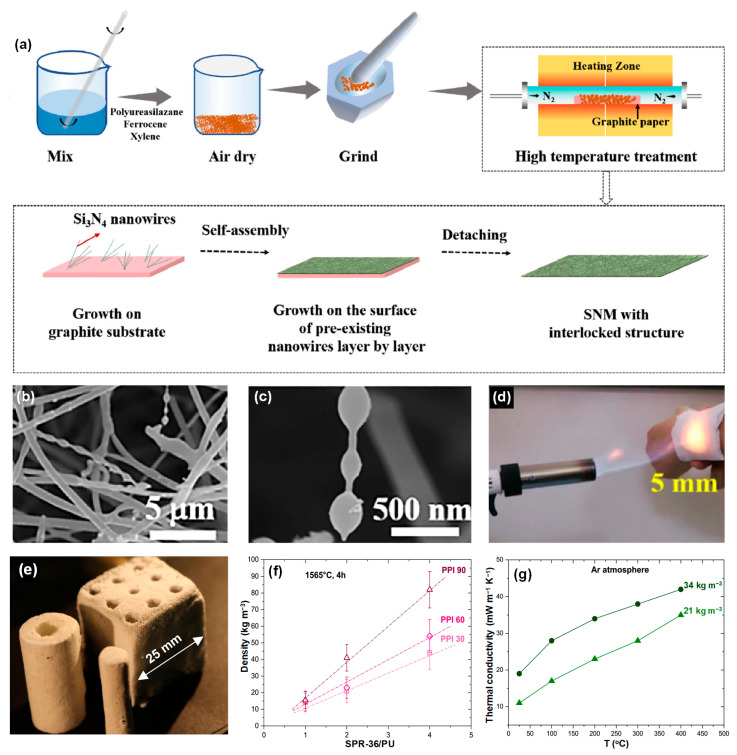
(**a**) Scheme illustrating the synthesis of Si_3_N_4_ nanowire membrane. (**b**,**c**) SEM images of the Si_3_N_4_ nanowires at different magnifications after heat treatment at 1300 °C. (**d**) Demonstration of fireproof and thermal insulation performance of Si_3_N_4_ nanowire membrane. (**e**) Si_3_N_4_ nanofelts with complex shapes. (**f**) The impact of relevant experimental parameters on the density of Si_3_N_4_ nanofelts. (**g**) Thermal conductivity of Si_3_N_4_ nanofelts. (**a**–**d**) Reproduced with permission [18]. Copyright 2020, Elsevier. (**e**–**g**) Reproduced with permission [87]. Copyright 2023, Elsevier.

Liu et al. [18] have successfully synthesized a Si_3_N_4_ nanowire membrane with exceptional flexibility, outstanding high-temperature resistance, and robust mechanical stability utilizing the precursor pyrolysis method. As illustrated in Figure 8a, the synthesized powders resulting from the drying of a composite precursor solution containing polyureasilazane, xylene, and ferrocene are deposited onto a U-shaped graphite paper substrate. Subsequently, the powders undergo a thermal treatment at 1300–1450 °C, transitioning into a Fe–Si–C–N liquid alloy phase. During this process, the Fe–Si–C–N liquid alloy incorporates N element from the surrounding N_2_ atmosphere, leading to the precipitation of Si_3_N_4_ nanowires. Subsequent to this, guided by gas flow dynamics, the Si_3_N_4_ nanowires self-organize into an intricately interlocked configuration. Finally, upon cooling of the furnace and subsequent detachment of the resultant products from the substrate, a Si_3_N_4_ nanowire membrane of significant structural integrity is obtained. The resultant Si_3_N_4_ nanowire membrane retains its intricately interlocked structure without experiencing substantial alterations in its macroscopic dimensions following heat treatment at 1300 °C (Figure 8b,c). The thermal conductivity of the Si_3_N_4_ nanowire membrane at room temperature was 0.056 W·m^−1^·K^−1^, which made it possible to protect the researcher’s arm from being burned by a 1000 °C flame (Figure 8d).

Moreover, Biesuz et al. [87] propose a rapid and straightforward approach for synthesizing Si_3_N_4_ nanofelts, that is, pyrolysis of polyurethane foam impregnated with liquid siloxane under N_2_ flow is carried out (Figure 8e). At a specified temperature, solid-phase SiO_2_ is carburized and reduced to SiO vapor, and then being converted to α-Si_3_N_4_ through the carbothermal reduction process. The resulting α-Si_3_N_4_ nanofelts exhibit exceptional porosity of 99.7% (10 kg·m^−3^) and demonstrate a low thermal conductivity of 0.011 W·m^−1^·K^−1^ in an argon (Ar) atmosphere (Figure 8f,g). This streamlined synthesis approach emphasizes the significant potential of these nanofelts for advanced thermal insulation applications.

#### 3.2.2. SiBN Fiber

SiBN fibers synergistically integrate the exceptional oxidation resistance inherent in Si_3_N_4_ fibers with the remarkable thermal stability characteristic of BN fibers, positioning it as a superior wave-transmitting reinforcing material in current applications. Tang et al. [88] successfully synthesized a novel high-performance SiBN fiber via the pyrolysis process of a carbon-containing polyborosilazane precursor. The resulting fiber exhibited remarkable thermal resistance, as evidenced by the sustained mechanical strength even after exposure to high temperatures of 1700 °C in a N_2_ atmosphere or 1400 °C in an O_2_ environment. Subsequently, Long et al. [19] studied the effect of boron content (3.56–6.81 wt%) on high-temperature stability of SiBN fibers. Higher concentrations of boron have been found to improve thermal durability of SiBN fibers at elevated temperatures, enhancing their suitability for use as microwave-transparent composites in demanding high-temperature environments. The synthesized SiBN fibers displayed complete amorphy, featuring a predominant presence of Si_3_N_4_ phase in conjunction with amorphous BN phases interconnected via Si–N–B networks. After being subjected to a temperature of 1600 °C, the SiBN fibers retained their original smooth surface and fully amorphous structure, leading to the preservation of their initial tensile strength to a significant extent. Moreover, as the temperature elevated from ambient conditions to 1200 °C, the SiBN fibers exhibited outstanding microwave-transmittance characteristics characterized by a minimal dielectric constant and loss.

Moreover, Long et al. [89] conducted a study examining the high-temperature oxidation behavior of SiBN fibers across a spectrum of boron concentrations, spanning from 1000 to 1400 °C under ambient air conditions. When the temperature above 1100 °C, SiBN fibers underwent oxidation, leading to the conversion of Si_3_N_4_ and BN phases into SiO_2_ and B_2_O_3_, respectively. The volatilization of molten B_2_O_3_ at high temperatures resulted in the retention of amorphous SiO_2_ on the fiber surface. Following oxidation, the penetration of molten B_2_O_3_ into the inner structure of the fiber facilitated its reaction with Si_3_N_4_, resulting in the emergence of hexagonal boron nitride (*h*-BN) nanoparticles and the development of a SiO_2_/BN layer. As a result, the oxidizing process yielded intricate oxidation layers consisting of two discernible concentric sublayers, each accompanied by two transitional sublayers.

#### 3.2.3. SiBCN Fiber

SiBCN fibers represent a new class of amorphous materials characterized by superior corrosion resistance, oxidation resistance, low density, high temperature resistance, commendable mechanical properties, and excellent wave penetration capabilities when compared to traditional Si_3_N_4_ and SiC binary fibers. Owing to the relatively low diffusion coefficients exhibited by non-oxide binary phases, such as SiC, BN, and Si_3_N_4_, alongside their thermal decomposition thresholds remaining below the corresponding melting points, the synthesis of this particular group of amorphous ceramics necessitates the utilization of the preceramic polymer pathway.

Wilfert et al. [90] utilized the pre-ceramic polymers derived from the aminolysis of dichloroboryl-methyl-trichlorosilyl-amine (DMTA) for the fabrication of SiBCN felts through electrospinning technique. Then, the fabricated precursor fibers were cured in an ammonia (NH_3_) environment rendering them infusible to avoid the melting during the subsequent process of pyrolysis. Finally, the polymeric fibrous materials were positioned in corundum crucibles and subjected to pyrolysis in quartz tubes under a N_2_ atmosphere at 1400 °C. Based on Thermogravimetric Analysis (TGA) investigations, the decomposition of SiBCN commences around 1700 °C and only a cumulative mass reduction of 14% was observed even at the terminal temperature of 1900 °C, highlighting the excellent thermal resistance of SiBCN felts.

Following this, Chen et al. [91] developed a new polyborosilazane precursor characterized by high purity and excellent formability. This was achieved through the utilization of tris(dichloromethylsilylethyl)borane as the primary raw material, in combination with boron trichloride and hexamethyldichlorosilane as crosslinking agents. After the electrospinning process and the follow-up pyrolysis treatment at 1000 °C under N_2_ atmosphere, the SiBCN nanofibers were obtained. However, in comparison to the SiBCN felts prepared by Wilfert et al. [90], the thermal stability of the resultant SiBNC nanofibers developed by Chen et al. has exhibited a slight decrease, with significant weight loss observed in the temperature range of 1500 to 1700 °C.

In a recent study, Tian et al. [21] integrated finely dispersed SiC nanograins into SiBCN fibers via a polymer-derived approach utilizing chemically modified polyborosilazane. Upon analysis, it becomes evident that the resultant SiBCN fibers were predominantly composed of amorphous SiBCN matrix, SiC nanograins, unbound carbon, and graphite-like BN(C) phase. The incorporated SiC nanograins could evidently enhance the tensile strength of SiBCN fibers. The remarkable resilience at extremely high temperatures can be traced to the existence of thermodynamically robust nanograins of SiC, alongside the encapsulation of these nanograins by the graphite-like BN(C) phase and the amorphous SiBCN matrix. This encapsulation mechanism further enhances the overall stability of the material system under extreme thermal conditions.

### 3.3. Carbide Ceramic Fiber Insulation Material

Carbide ceramic fibers exhibit outstanding properties, including being lightweight, strong, and having a high modulus. They also have exceptional resistance to high temperatures, corrosion, and oxidation. These fibers find extensive use in aerospace, nuclear, and other high-temperature sectors. They are used for insulation in rocket engines, lining high-temperature pipes, and manufacturing high-temperature electronic components. Currently, carbide ceramic fibers that are commonly prepared include silicon carbide (SiC) and zirconium carbide (ZrC).

#### 3.3.1. SiC Fiber

**Figure 9:**
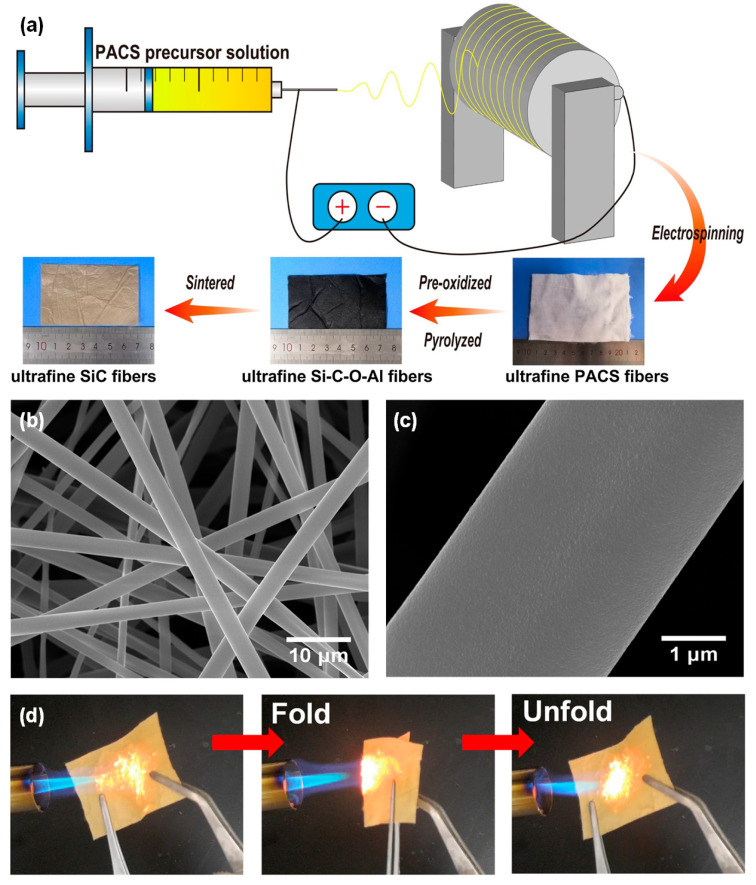
(**a**) Diagrammatic drawing of the SiC fiber membrane. (**b**,**c**) SEM images of the resulting SiC fibers at different magnifications. (**d**) Flexible demonstration of SiC fibers in a blowtorch flame at 1100 °C. (**a**–**d**) Reproduced with permission [96]. Copyright 2022, Elsevier.

SiC fiber has gained increasing attention due to its high-temperature stability, corrosion resistance, thermal conductivity, and semiconductor characteristics that make it applicable in demanding environments [92]. Currently, the precursor conversion method is one of the primary techniques for preparing SiC fibers. This method involves using organic polymers that contain carbon and silicon as precursors [93,94,95]. These precursors are then spun into fibers using either melt spinning or electrospinning techniques. Following high-temperature pyrolysis, SiC fibers with diameters spanning from tens of nanometers to several micrometers are successfully synthesized. Polycarbosilane (PCS) is acknowledged as a highly suitable precursor for the fabrication of SiC fibers. Chen et al. [96] employed a hybrid approach, combining electrospinning with the polymer-derived ceramic method, to fabricate a pliable and ultrafine polycrystalline SiC fibrous membrane, as presented in Figure 9a–c. The resulting SiC fibers exhibit impressive flexibility, outstanding oxidation resistance, and exceptional high temperature thermal stability (Figure 9d). The fibers experienced only a 1.52% weight loss when subjected to temperatures up to 1900 °C in an Ar environment and a mere 4.1% weight gain when exposed to temperatures up to 1500 °C in an air atmosphere. The ultrafine SiC fibers have a crystalline structure consisting of SiC crystals with an average grain size of about 100 nm. They have a slight excess of carbon, as indicated by a C/Si ratio of 1.06, and a negligible presence of oxygen elements, constituting only 0.47 wt%. These compositional characteristics synergistically contribute to the remarkable thermal resistance exhibited by the fibers.

**Figure 10:**
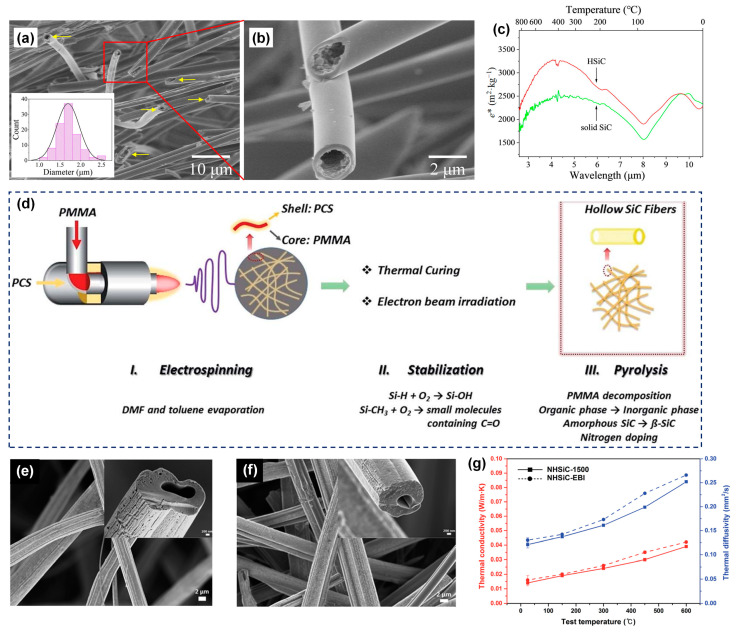
(**a**,**b**) SEM images of hollow-structured SiC fibers, inset histogram showing the diameter distribution of the fibers. The arrows indicate the location of the hollow SiC fibers. (**c**) The effective extinction coefficients of the two types of fiber mats. (**d**) Schematic diagram of the preparation of N-doped hollow SiC fiber mats. SEM images of the resulting N-doped hollow SiC fiber prepared through (**e**) thermal decomposition at 1500 °C and (**f**) electron beam irradiation. (**g**) The thermal conductivity and thermal diffusivity of these two fibers within the temperature range of 0–600 °C. (**a**–**c**) Reproduced with permission [26]. Copyright 2019, Elsevier. (**d**–**g**) Reproduced with permission [97]. Copyright 2017, RSC.

To enhance the thermal insulation performance of SiC fibers, Tian et al. [26] developed hollow-structured SiC ultrafine fibers using the emulsion electrospinning technique, followed by pre-oxidation and pyrolysis treatment. The resulting SiC fibers were consistently hollow with a narrow diameter distribution ranging from 1.2 to 2.5 μm and a wall thickness measuring 250 nm (Figure 10a,b). The extinction coefficient of hollow SiC fibers in the infrared region of 2.5 to 6 μm increases by approximately 30% when compared to the solid SiC fiber mat (Figure 10c). At a temperature of 800 °C, the thermal conductivity of hollow SiC fiber measures 0.1049 W·m^−1^·K^−1^, signifying a considerable reduction compared to that of solid SiC fiber, which registers at 0.3631 W·m^−1^·K^−1^. This indicates the crucial role of the hollow structures in improving the fibers’ thermal insulation performance. In contrast, Liu et al. [97] utilized the co-axial electrospinning technique to create N-doped hollow SiC fibrous mats, as demonstrated in Figure 10d. The shell layer was composed of PCS solution while the core layer consisted of poly(methyl methacrylate) (PMMA) solution. To preserve the fiber morphologies during the pyrolysis process, the precursor fibers were crosslinked through thermal oxidation in an atmospheric environment of air. Afterward, the crosslinked fibers underwent pyrolysis at varying temperatures in a N_2_ atmosphere to prepare N-doped hollow SiC fibers (Figure 10e). Alternatively, the as-spun core–shell fibrous mats fibers were cured through electron beam irradiation (EBI) (Figure 10f). The introduction of nitrogen doping led to a noteworthy reduction in thermal conductivity for the hollow SiC fibers, with the N-doped variant exhibiting a value of 0.026 ± 0.013 W·m^−1^·K^−1^ at 25 °C, as compared to the 0.138 W·m^−1^·K^−1^ observed for the undoped fibers at the same temperature (Figure 10g). This observation underscores the significant impact of nitrogen doping on enhancing thermal insulation properties.

To enhance the high-temperature resistance and thermal insulation capabilities even further, Zhang and co-workers [98] introduced zirconium precursor into the spinning solution. After the electrospinning process and subsequent treatment at 1300 °C in an Ar atmosphere, ZrO_2_/SiC nanofibrous membranes were obtained. TGA indicated negligible weight loss upon heating the ZrO_2_/SiC nanofibrous membranes to 1400 °C, a temperature notably surpassing the threshold necessary for SiC nanofibers alone, which is approximately 1100 °C. It shows that the introduction of ZrO_2_ has a great promoting effect on the temperature resistance of the membranes. The addition of ZrO_2_ phases with low thermal conductivity, SiC phases with infrared shielding properties, and residual carbon within the ZrO_2_/SiC composite nanofibers leads to a decrease in thermal conductivity. The thermal conductivity was measured to be 0.032 W·m^−1^·K^−1^ at room temperature and 0.217 W·m^−1^·K^−1^ at 1300 °C. In addition, hollow SiO_2_ nanospheres were incorporated as a pore-forming agent into the SiC fiber matrix to construct dense cellular structures [99]. Due to the closed pore structure and the existence of multiphase interfaces, the resulting SiO_2_/SiC fibrous membranes exhibit low thermal conductivity (0.026 W·m^−1^·K^−1^) and high temperature resistance (1400 °C).

#### 3.3.2. ZrC Fiber

ZrC fibers exhibit exceptional physical and chemical properties, including high hardness, elevated melting point, thermal shock resistance and superior mechanical strength. As a prominent component of ultrahigh temperature ceramics, ZrC fibers have extensive applications in the aerospace industry, particularly in hypersonic aircraft and related fields. Currently, the main methods for producing ZrC fibers include carbothermal reduction, mechanical alloying, self-propagating high-temperature synthesis, and electrospinning [100], among which electrospinning is considered to be an efficient and facile approach. In 2008, Cui and co-workers [101] successfully fabricated ZrC nanofibers by electrospinning and annealing at 1600 °C for 2 h in an Ar atmosphere. The ZrC crystalline phase started to form initially at 1500 °C. Subsequently, Li et al. [102] found that the carbon source has a great influence on the fiber morphologies by modulating the kinetics of the carbothermal reduction process.

Ge et al. [103] incorporated ZrC and SiC into uniform fibers to resist the creep of SiC fibers. By subjecting a preceramic precursor of polyzirconocenecarbosilanes (PZCS) to melt spinning, electron beam curing, and pyrolysis processes, a continuous ceramic fiber with SiC-ZrC binary phases was successfully fabricated. After subjecting the green fiber cured by the electron beam to decarbonization in a hydrogen atmosphere at 1000 °C, the resulting fiber was calcined in an Ar environment at 1600 °C. This process generated stoichiometric α-SiC and ZrC phases within the fibers. ZrC particles with average diameters in the range of 15 to 20 nm were generated and uniformly distributed in the SiC fiber matrix.

Afterward, Wang and co-workers [22] prepared ZrC fibers through centrifugal melt-spinning using zirconium-containing polymers as precursor. The synthesized precursor solution was placed into the melting chamber where rapid melting occurred. The molten material was then extruded through the spinneret orifice, facilitated by centrifugal forces, and formed into ZrC precursor fibers that were collected using a concentric barrel arrangement. Then, the fabricated precursor fibers were annealed at 1500 °C for 2 h. Compared with the previously reported ZrC fibers, the structure of the fibers prepared by centrifugal melt-spinning is more compact.

## 4. 3D Ceramic Fiber Aerogels

Although the traditional 2D micro-nanofiber thermal insulation material exhibits good high-temperature thermal insulation performance, it still faces challenges such as limited thickness, low porosity and thermal insulation performance that require further improvement. Compared to 2D fibrous membranes, 3D fiber aerogel materials offer advantages such as controllable size, high porosity, and a high degree of pore zigzagging. Hence, they possess broad potential applications in areas such as thermal insulation, heat retention, and acoustical absorption. A wide variety of ceramic fibers have been constructed into 3D fibrous aerogels by a variety of preparation methods, as summarized in Table 2. Considering that the preparation method of fiber aerogels has a decisive influence on their spatial structure, which in turn directly affects their high-temperature thermal insulation performance, we classify and review 3D ceramic fiber aerogels according to different preparation methods (direct spinning, template, sol-gel impregnation, vacuum filtration molding, freeze drying, and stacking) in the following sections.

### 4.1. Direct Spinning Method

**Figure 11:**
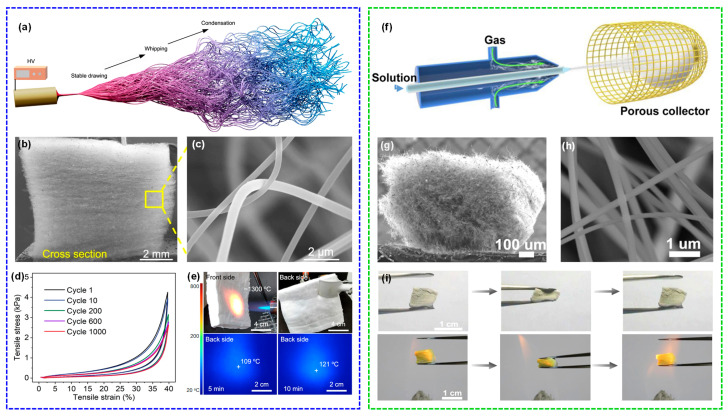
(**a**) Schematic diagram of direct fabrication of 3D ceramic fiber aerogels by electrospinning. (**b**,**c**) SEM images of 3D interwoven nanofiber aerogel. (**d**) A 1000-cycle tensile test conducted at a tensile strain of 40%. (**e**) The resulting nanofibrous aerogels heated by a burner flame at 1300 °C for 10 min. (**f**) Scheme depicting the fabrication of 3D fibrous aerogel using blow-spinning technique. (**g**,**h**) SEM image of TiO_2_ fibrous sponge at different magnifications. (**i**) The compression recovery process of TiO_2_ fibrous sponges at ambient conditions and under alcohol lamp flame. (**a**–**e**) Reproduced with permission [27]. Copyright 2022, Nature Portfolio. (**f**–**i**) Reproduced with permission [28]. Copyright 2017, AAAS.

The process of depositing fibers directly into bulk fiber material in the receiver by electrospinning, blowing, and other spinning technologies is called direct spinning. Cheng et al. [27] proposed a three-dimensional reaction electrospinning process to directly fabricate a ceramic nanofibrous aerogel through manipulating the sol jet coagulation during its trajectory. Low quantities (0.1 wt%) of high molecular weight polymer were added into the synthesized precursor sol to change the jet injection mode into a multi-jet spinning, as demonstrated in Figure 11a. Through adjusting the vertical displacement of the spinning nozzle and collector, they were able to obtain a 3D interwoven nanofiber aerogel precursor through interknitting the crimped nanofibers (Figure 11b,c). The fabricated ceramic aerogels exhibited good tensile and torsion resistance as well as superior compressibility (Figure 11d). Furthermore, the resultant ceramic aerogels demonstrated exceptional thermal stability across a wide temperature range from −196 to 1400 °C, accompanied by a notably low thermal conductivity of 0.0228 W·m^−1^·K^−1^ at room temperature and robust fire resistance (Figure 11e). Dong et al. [121] developed a lamellar-structured ZrO_2_-TiO_2_ fiber sponge by a coaxial electrospinning technique followed by the calcination process. The precursor solutions for ZrO_2_ and TiO_2_ were prepared separately using the synthesized polyaceticzirconium and polyacetylacetonatotitanium, respectively. The solutions were injected into the core and shell channels of the coaxial needle. The ZrO_2_-TiO_2_ fiber sponge was designed to be highly compressible and resilient in the temperature range of −196 to 1200 °C. The ZrO_2_-TiO_2_ fiber sponge exhibited a low thermal conductivity of 0.027 W·m^−1^·K^−1^ and a high near-infrared reflectivity of over 97%, rendering it highly suitable for lightweight insulation applications requiring high temperature resistance.

Wang et al. [28] produced ceramic nanofiber sponges, including TiO_2_, ZrO_2_, YSZ, and BaTiO_3_, using a solution blow-spinning process. An air-permeable cage-like container was used as the fiber collector, as exhibited in Figure 11f–h. The prepared ceramic nanofiber sponge can recover rapidly after compression of more than 20% strain at room temperature and 400 °C, and maintain good recovery after compression at 1300 °C (Figure 11i). The ZrO_2_ nanofiber sponge exhibited a low room-temperature thermal conductivity of 0.027 W·m^−1^·K^−1^, benefitting from its high porosity and porous cellular structures. Afterwards, Zhu et al. [112] prepared elastic layered mullite fiber sponge with high compression and high temperature resistance using the same strategy. The fabricated mullite fiber sponge could maintain 94.34% of the initial maximum stress after 100 compression cycles with ~50% strain, demonstrating the remarkable compressibility. In addition, the mullite fiber sponge exhibits excellent compressive elasticity within a broad temperature interval extending from −196 to 1200 °C and a thermal conductivity of 0.0265 W·m^−1^·K^−1^ at 40 °C.

To enhance the heat-insulating capabilities of ceramic fiber sponge, Xu et al. [107] used blow spinning and atom layer deposition (ALD) to prepare PVP/Al_2_O_3_ nanofibrous aerogel. Subsequently, Al_2_O_3_ nanotube aerogels were obtained by calcination treatment after removing the sacrificial template PVP. The Al_2_O_3_ nanotube aerogels demonstrated high resilience, exhibiting only 20% plastic deformation after 100 compression cycles at approximately 60% strain. The Al_2_O_3_ nanotube aerogels showed remarkable elasticity even after being subjected to a temperature of 900 °C for 2 h. Additionally, they displayed a low room-temperature thermal conductivity of 0.022 W·m^−1^·K^−1^.

The ceramic nanofiber sponge material prepared by the direct spinning method exhibits certain compression and rebound characteristics. However, irregular shape of the collected fiber material, difficulty in controlling the thickness, and poor bonding between fibers are some of the problems that arise due to the need to use a specific receiver to collect the fiber material.

### 4.2. Template Method

To ensure the controllable shaping of the prepared 3D ceramic nanofiber aerogel, 3D fiber blocks with regular shapes are used as templates. Ceramic components are deposited on the surface of the block by chemical vapor deposition (CVD) or ALD, and then the template is removed to obtain a 3D ceramic fiber aerogel. Su et al. [119] employed a porous carbon foam (CF) as a sacrificial template and coated SiC layer onto the CF skeleton through CVD process. SiC foam was obtained after removing the carbon matrix in the bulk by heat treatment in a muffle furnace at 800 °C for 10 h. Using the CVD technique, SiC nanowires (SiC_nw_) were subsequently deposited into the SiC skeleton resulting in the synthesis of SiC_nw_@SiC foam with a double network structure. The SiC_nw_@SiC foam demonstrated outstanding thermal insulation performance at high temperatures, with a thermal conductivity of 0.304 W·m^−1^·K^−1^ at 1200 °C. Additionally, the compressive strength was measured at 1.53 MPa, indicating the composite foam’s potential for use in harsh environments.

**Figure 12:**
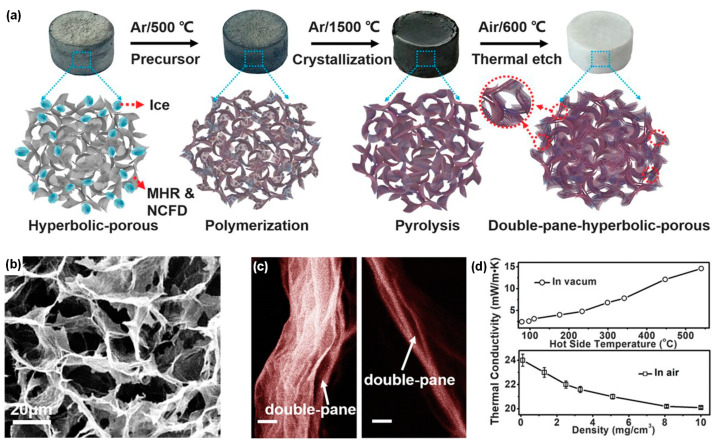
(**a**) Flowchart of chemical vapor deposition process for generating hyperbolic ceramic aerogels with a dual-pane structure. (**b**,**c**) SEM images of the obtained dual-paned aerogel. (**d**) Thermal conductivity of hBNAGs in different atmospheres. (**a**–**d**) Reproduced with permission [30]. Copyright 2019, AAAS.

Xu and co-workers [30] used the freeze-dried graphene aerogel as a sacrificial template to synthesize hexagonal boron nitride aerogels (hBNAGs) and β-silicon carbide aerogels (βSiCAGs) by a catalyst-free CVD method and subsequent annealing treatment to etch the graphene template (Figure 12a). The synthesized ceramic aerogels possessed hyperbolic-patterned macrostructures with a negative Poisson’s ratio of −0.25 and superelasticity with a maximum strain of 95% (Figure 12b,c). Moreover, the resultant aerogels showcased a combination of characteristics including ultralow density (reaching as low as 0.1 mg·cm^−3^), low thermal conductivity of approximately 0.02 W·m^−1^·K^−1^ in air (Figure 12d), robust thermal stability even under rapid thermal shocks (up to ~275 °C·s^−1^), and sustained performance under prolonged exposure to high temperatures (900 °C in air and 1400 °C in vacuum). This approach is versatile and could be used to prepare various varieties of ceramic fibrous aerogels. Thereafter, Guo et al. [29] prepared a foam of single-crystal Si_3_N_4_ nanowires by growing them in situ on a green body composed of activated carbon particles, followed by heat treatment to remove the activated carbon.

### 4.3. Sol-Gel Impregnation Method

Conventional ceramic aerogels are generally in a state of particle accumulation, with high static air retention and low thermal conductivity. However, they have defects such as high brittleness and poor vibration resistance, which limit their application. In addition, the fusion between the particles would easily cause the structure to collapse at high temperature, so the high temperature resistance of the granular ceramic aerogel is poor. The introduction of ceramic fibers as reinforcing phases in aerogels can improve the aerogel skeleton and bridging, thereby producing ceramic aerogel composites with improved mechanical properties and excellent thermal insulation properties.

He et al. [122] first used a molding press method to construct the mullite fiber green body, and then impregnated it into the ZrO_2_-SiO_2_ sol to form wet gel. After aging and supercritical drying, the aerogels/fibrous ceramic composite was obtained. The as-synthesized composite aerogel exhibited a remarkable compressive strength of up to 1.05 MPa and a low room-temperature thermal conductivity of 0.0524 W·m^−1^·K^−1^. Moreover, the thermal conductivity in the temperature range of 500 to 1200 °C was 0.082 to 0.182 W·m^−1^·K^−1^, highlighting its superior thermal insulation performance over a wide temperature range. Subsequently, they developed large-scale fiber/aerogel composites using ZrO_2_ fiber felt as the skeleton and ZrO_2_-SiO_2_ aerogels as the obturator by vacuum impregnation, aging, and supercritical drying [123]. The ZrO_2_ fiber/ZrO_2_-SiO_2_ composite aerogels demonstrated significantly reduced thermal conductivity, measuring 0.0341 W·m^−1^·K^−1^ at ambient temperature and ranging from 0.046 to 0.096 W·m^−1^·K^−1^ within the temperature span of 500 to 1100 °C.

**Figure 13:**
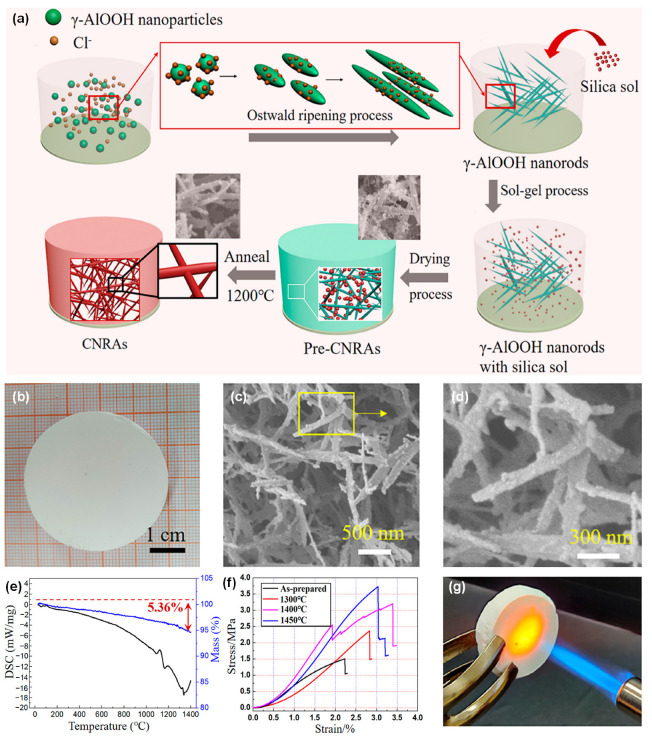
(**a**) Scheme illustrating the preparation procedure of ceramic nanorod aerogels. (**b**) Digital photograph and (**c**,**d**) SEM images of the synthesized ceramic nanorod aerogels. (**e**) Thermogravimetric-differential scanning calorimetry (TG-DSC) analysis for ceramic nanorod aerogels. (**f**) Compression stress–strain curves of the aerogels before and after heat treatment. (**g**) The resulting ceramic nanorod aerogels heated by a burner flame at 1300 °C without damage. (**a**–**g**) Reproduced with permission [31]. Copyright 2021, ACS.

Zhang et al. [31] proposed a facile and scalable approach for fabricating ceramic nanorod aerogels featuring hierarchical macroporous and mesoporous architectures. This approach involves the controlled assembly of Al_2_O_3_ nanorods and SiO_2_ nanoparticles (Figure 13a–d). The subsequent high-temperature annealing treatment ensures the strong Si-O-Al bond between adjacent nanorods, giving the aerogel a high compressive strength of 1.5 MPa, extraordinary fire resistance and a temperature resistance of up to 1400 °C (Figure 13e–g). Furthermore, the ceramic nanorod aerogels demonstrated low thermal conductivity of 0.026 W·m^−1^·K^−1^ at 25 °C and 0.089 W·m^−1^·K^−1^ at 1200 °C. The synthesized ceramic nanorod aerogels demonstrated a combination of characteristics, including heightened thermal stability, reduced thermal conductivity, increased mechanical resilience, light weight, superior thermal insulation capability, and scalability in fabrication. These properties collectively underscore their significant potential for application in extreme environments.

### 4.4. Vacuum Filtration Molding Method

The vacuum filtration molding process involves mixing the fibers, binders, and solvents to create a uniformly dispersed slurry. This slurry is then poured into a porous mold and subjected to vacuum filtration molding under the pumping force of a vacuum pump. The resulting green body are dried and then subjected to high-temperature sintering to produce porous structured ceramic fibrous aerogel.

Zhang et al. [110] used mullite fiber as a skeleton, polyethyleneimine (PEI) as a dispersant, polyacrylamide (PAM) as an adsorbent, SiC and B_4_C as inorganic binders, and starch as an organic binder to prepare the fibrous slurry. The well-stirred slurry was filtrated using a vacuum pump and an initial pressure of 160 kPa was applied on the head during the filtration process. After vacuum filtration treatment, the wet fiber network was dried and then calcined at 1400 °C for 1 h to remove the organic binder and form bonding points between the fibers. The resulting ceramic fibrous aerogel exhibited a high compressive strength of 2.1 MPa with a porosity of 87.3%. Moreover, the thermal conductivity of the fibrous porous mullite ceramics at room temperature was 0.093 W·m^−1^·K^−1^. Afterwards, Zhang’s group [124] created mullite-ZrO_2_ ceramics with a lower thermal conductivity of 0.052 W·m^−1^·K^−1^ using the same procedure.

Subsequently, Dong et al. [125] prepared fiber slurry by mechanically mixing mullite fiber, silicon resin, and solvent, followed by vacuum filtration treatment. The resulting green bodies were subjected to a heating and crosslinking process, and then calcined at various temperatures to obtain the molded fiber block. After undergoing vacuum filtration treatment, a portion of the binder becomes trapped between the fibers, resulting in the formation of bonding points after calcination treatment. The resulting mullite fibrous aerogel exhibited a high compressive strength of 1.21–1.58 MPa and a high rebound-resilience of 70–85%. The minimum thermal conductivity of the constructed fibrous ceramics was 0.083 W·m^−1^·K^−1^ (room temperature).

**Figure 14:**
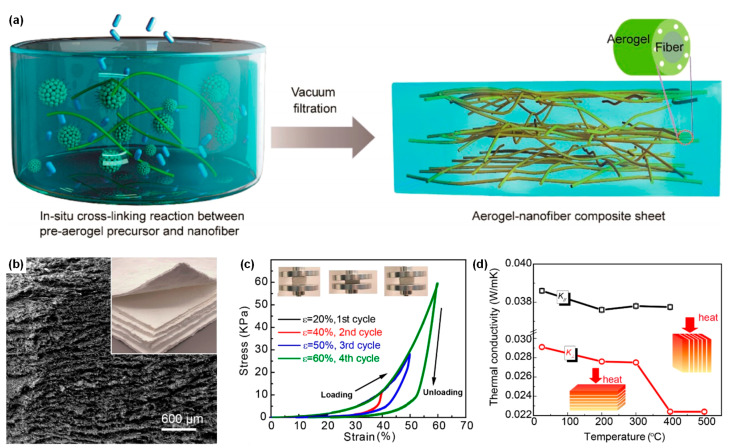
(**a**) Schematic illustration of the manufacturing process of fibrous aerogels. (**b**) SEM image and digital photo of the resultant stacked aerogel sheet. (**c**) Compressive strain versus stress curves for the fibrous aerogels after sintering at 400 °C. (**d**) The relationship between thermal conductivity in different directions and the sintering temperature. (**a**–**d**) Reproduced with permission [32]. Copyright 2020, ACS.

Moreover, An and co-workers [32] uniformly dispersed the aluminoborosilicate nanofiber in water with SiO_2_ aerogel precursor and the dried SiO_2_ aerogel powder to prepare fiber slurries, respectively (Figure 14a). The obtained slurries were then treated by vacuum filtration process to prepare the resultant aerogel composite. The resulting lamellar-structured aerogel composite demonstrated high flexibility, outstanding recoverability with a strain recoverable compressibility larger than 50%, and excellent fatigue resistance (Figure 14b,c). The interfacial interaction between aluminoborosilicate nanofibers and SiO_2_ aerogels plays a major role in the anisotropic thermal insulation of the obtained aerogel, contributing to the low thermal conductivity of 0.0224 W·m^−1^·K^−1^ (Figure 14d).

### 4.5. Freeze-Drying Method

The methods described above have proven to be efficient approaches for preparing ceramic fiber aerogels. However, the resulting ceramic materials still have weak thickness controllability and relatively high-volume density. Alternatively, the freeze-drying method involves low-temperature freezing to solidify the precursor solution. The solidified solvent crystals are then sublimated by vacuum drying, preserving the shape and structure of the sample. Thus, the size, shape, and volume density of the ceramic fiber aerogels could be precisely regulated.

**Figure 15:**
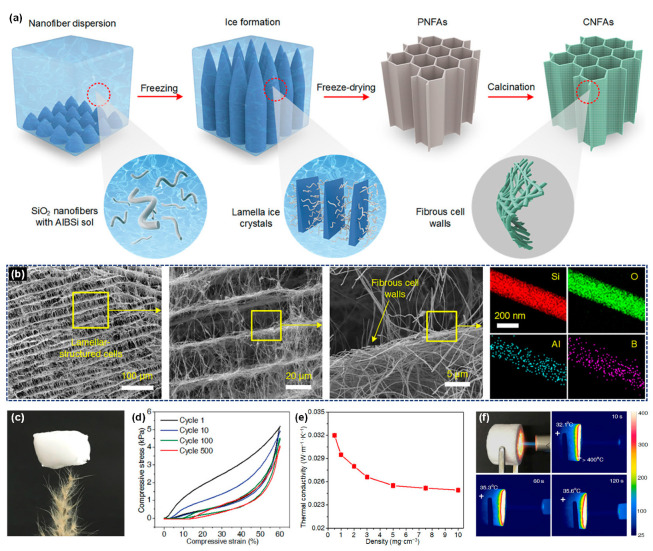
(**a**) Scheme illustrating the fabrication of CNFAs. (**b**) SEM and STEM-EDS images of CNFAs. (**c**) The photo depicts CNFAs with a bulk density of 0.15 mg·cm^−3^ positioned at the extremity of a feather. (**d**) A compressive fatigue test consisting of 500 cycles under strain of 60%. (**e**) The relationship between the thermal conductivity of CNFAs and their bulk density. (**f**) Optical and infrared images of CNFAs under the torch flame for 120 s. (**a**–**f**) Reproduced with permission [34]. Copyright 2018, AAAS.

Si et al. [34] manufactured lamellar-structured nanofibrous ceramic aerogels (CNFAs) with super-lightweight properties using flexible SiO_2_ nanofibers as skeleton and aluminoborosilicate (AlBSi) sol as high-temperature adhesive (Figure 15a–c). The resulting CNFAs exhibited structural robustness and high resilience; however, the aerogels with a disordered structure or an unbonded network derived from identical precursors exhibited structural collapse under significant compressive loads, demonstrating that cellular structures and stable bonding between fibers play an important role in elastic recovery. After 500 load-unload cycles at 60% strain, the plastic deformation was 12%, indicating the good long-term cycling performance of the resulting CNFAs (Figure 15d). The CNFAs exhibited a low thermal conductivity of 0.025 W·m^−1^·K^−1^ and a temperature resistance of 1100 °C (Figure 15e,f). Subsequently, other species of ceramic fiber aerogel have been developed using the freeze-drying method, such as SiC@SiO_2_ nanowire aerogel, mullite nanofibrous aerogels, Si_3_N_4_ fiber aerogel, TiO_2_ nanofiber aerogel, BN aerogels, etc.

The aforementioned aerogels are generally produced by high-temperature calcination to create bonding points between fibers. In this process, ceramic fibers are easily transformed from amorphous to crystalline state or secondary growth of grains. The brittle crystalline structure limits further improvements of mechanical properties. For this reason, Wang et al. [126] fabricated a biomimetic SiO_2_ nanofiber aerogel exhibiting superelasticity through the integration of flexible SiO_2_ nanofibers and a rubber-like Si-O-Si bonding network. The resulting ceramic aerogels achieve in situ fiber bonding during the freeze-drying process, and their superelasticity could be achieved without further calcination, avoiding the brittle structure caused by secondary high-temperature calcination. They found that the elastic restitution of ceramic aerogels prepared with trimethoxymethylsilane (MTMS) as the silicon source was superior to that of aerogels prepared with tetraethyl orthosilicate (TEOS)and dimethoxydimethylsilane (DMDMS) as the silicon source. The resulting ceramic fiber aerogel demonstrated robust fatigue resistance, enduring over a million compressions, and the aerogels exhibited low thermal conductivity of 0.024 W·m^−1^·K^−1^ with a temperature resistance of 1100 °C.

To further enhance the thermal insulation capabilities of the ceramic fibrous aerogels, Dou et al. [127] used SiO_2_ nanofibers and SiO_2_ nanoparticle aerogels as building blocks for the construction of hierarchical cellular structured ceramic nanofiber aerogel. The SiO_2_ fibers and SiO_2_ aerogel particles form a stable Si-O-Si bond structure that ensures the mechanical stability of the aerogel material in the process. The prepared composite aerogel has excellent fire resistance and high temperature thermal insulation properties with a thermal conductivity of 0.02327 W·m^−1^·K^−1^.

### 4.6. Stacking Method

Although a ceramic fiber aerogel with certain resilience can be prepared by the above freeze-drying method, the internal fibers of the aerogel bear the external stress in a point-to-point contact manner, which is difficult to endure intense mechanical forces or heat flow impact in practical application. Therefore, the aerogel will cause structural collapse and strength degradation under external force or long-term exposure to high temperature, which will seriously affect its stability. The ceramic fiber layers are stacked in parallel, which could largely withstand the compressive deformation and stress.

Su et al. [115] first prepared the paper-like *α*-Si_3_N_4_ nanoribbon sheets using siloxane xerogels as raw materials, and then the *α*-Si_3_N_4_ papers were annealed at 1000 °C in air to form thin SiO_2_ shell on the surface of nanoribbons. Afterward, the treated film was assembled layer by layer and the aerogel bulk was obtained after hot pressing heating treatment at 1200 °C. The resulting amorphous SiO_2_ layer would act as a binder to create adhesion points between the nanobelts during the hot-pressing heat treatment. The resulting *α*-Si_3_N_4_ nanobelt aerogel has adjustable bulk density ranging from 1.8 to 9.6 mg·cm^−3^. The *α*-Si_3_N_4_ nanobelt aerogel exhibited resilient compressibility with a recoverable strain of 40 to 80% and a low thermal insulation of 0.029 W·m^−1^·K^−1^ with a temperature resistance of 1200 °C in ambient air. Although a certain bonding structure is established between the nanobelts after heat treatment, there is still a lack of support structure between the layers, and the interaction force between the layers is weak, resulting in the problem that the material is still easy to peel off between the layers.

To further tackle this problem, Zhang et al. [114] constructed a lamellar multiarch structured ceramic nanofiber aerogel by a three-dimensional construction method combining impregnation stacking and freeze-drying process, using flexible ZrO_2_-Al_2_O_3_ nanofibrous membranes as the building blocks and aluminum dihydrogen phosphate (Al(H_2_PO_4_)_3_) as the binder. The ZrO_2_-Al_2_O_3_ nanofibrous aerogels that have been produced exhibited a comprehensive set of properties, including rapid strain recovery up to 90%, compressive strength in excess of 1100 kPa at 90% strain, remarkable fatigue resistance, and consistent superelasticity over a range of temperatures. In addition, the ZrO_2_-Al_2_O_3_ nanofibrous aerogels demonstrated a relatively low thermal conductivity of 0.0322 W·m^−1^·K^−1^ and high temperature resistance of up to 1300 °C.

**Figure 16:**
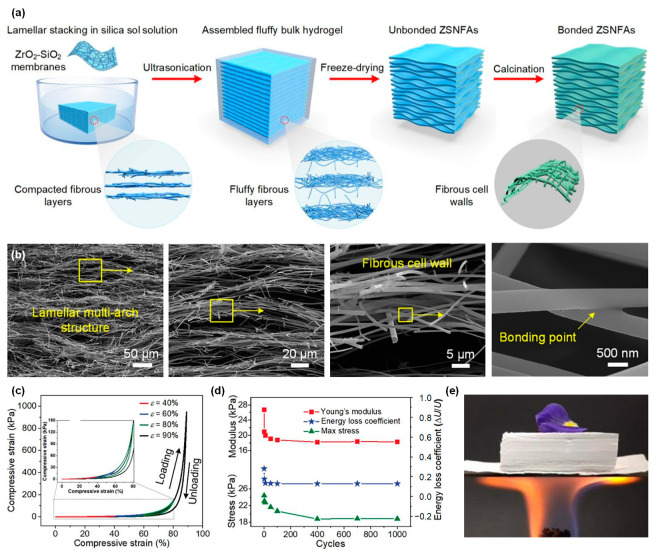
(**a**) Sketch presentation for the construction of ceramic nanofiber aerogel with layered multi-arch structures. (**b**) SEM images of the resulting lamellar multiarch structured fiber aerogel at different magnifications. (**c**) Compression cycle curves under various strains. (**d**) Relationship between compression cycles and Young’s modulus, energy loss coefficient, and maximum stress. (**e**) The ZrO_2_-SiO_2_ nanofibrous aerogels could protect the flowers from burning out. (**a**–**e**) Reproduced with permission [35]. Copyright 2022, Elsevier.

To solve the problem of poor fatigue life caused by large energy dissipation of the above prepared ceramic nanofiber aerogel, Zhang et al. [35] immersed the flexible ZrO_2_-SiO_2_ nanofibrous membranes with a predetermined size into the SiO_2_ sol solution, and then prepared an aerogel with a fluffy layered arched honeycomb structure by ultrasonication, stacking, freeze-drying, and calcination, as displayed in Figure 16a,b. The developed ZrO_2_-SiO_2_ nanofibrous aerogel demonstrated constant superelasticity across a temperature range of −196 to 1100 °C and a high compressive strength of 950 kPa at 90% strain (Figure 16c). In particular, the resulting aerogel exhibited a low energy loss coefficient of 0.28 and exceptional fatigue resistance, maintaining nearly zero plastic deformation even after undergoing 1000 compression-recovery cycles (Figure 16d). Moreover, the aerogel possessed a low room-temperature thermal conductivity of 0.0268 W·m^−1^·K^−1^. Subsequently, the thermal insulation performance of the ZrO_2_-SiO_2_ nanofibrous aerogel was further improved through employing the silicon particle aerogel into the matrix. On the basis of maintaining the excellent elastic recovery and compression fatigue resistance of aerogels, the thermal conductivity of aerogels was further reduced to 0.024 W·m^−1^·K^−1^. As demonstrated in Figure 16e, the flowers could be protected from burn-out by ZrO_2_-SiO_2_ nanofibrous aerogels, revealing their extraordinary thermal insulation performance.

## 5. Challenges and Future Trends

Although significant progress has been made in the development of ceramic micro-nanofiber insulation materials, there are still several challenges that need to be addressed before ceramic micro-nanofibers can be applied in practice:

1. Although some ceramic micro-nanofibers can be transformed from brittle to flexible by adjusting the sol-gel process, calcination process parameters and ion doping, there is still no flexible mechanism to explain this transformation process. Therefore, in situ mechanical devices can be used to study the structural transformation of ceramic micro-nanofibers during the stress process. With the help of molecular dynamics and finite element simulation, the models of single fiber and fiber assembly are constructed to simulate the stress process of ceramic fiber materials, so as to provide a scientific explanation of the flexible mechanism of ceramic fiber, and to provide guidance for the preparation of other kinds of high-temperature resistant flexible ceramic micro-nanofiber materials.
2. At present, the mechanical strength of ceramic micro-nanofibrous materials is relatively low, which makes it challenging to fulfill the needs of practical applications. Therefore, it is of vital importance to further improve the mechanical properties of ceramic micro-nanofibrous materials. The tensile strength of a single fiber can be enhanced by various means, such as controlling the crystallinity of the ceramic fiber, changing the direction of grain orientation, reducing micro defects in the fiber, and densifying the fiber. In addition, the mechanical properties of fiber aggregates can be improved by creating adhesion points between fibers, changing the direction of fiber accumulation, and increasing the degree of entanglement between fibers.
3. Presently, the thermal conductivity of the ceramic micro-nanofiber insulation material has been reduced to some extent. However, the material still exhibits high thermal conductivity at high temperatures. In addition, the currently manufactured ceramic oxide nanofibers are typically polycrystalline. When used under high temperature conditions, there is secondary growth of grains, which further reduces the strength of the material. Therefore, it is necessary to improve the high-temperature insulation performance and resistance of the material. By directly processing ceramic materials with low solid thermal conductivity and high infrared reflectivity into 2D flexible ceramic micro-nanofibrous membranes or 3D aerogels. Alternatively, high reflectivity or high infrared shielding coatings are sprayed onto the surface of existing ceramic fiber materials. These approaches can be helpful in improving the high-temperature thermal insulation performance of materials.
4. Electrospinning and air-jet spinning technologies are the primary methods for producing ceramic micro-nanofibers, and industrial equipment is now available. Despite these advances, the efficiency and yield of these technologies remain lower than those of conventional ceramic fibers, requiring further improvements in the stability of material morphology and structure. In addition, the development of ceramic fiber aerogels is still in the experimental phase, with a relatively long process flow, indicating the need for optimization in the production process. Therefore, it is necessary to optimize the production process and transform the production equipment based on the existing research and existing equipment to realize the industrial production of ceramic micro-nanofibrous materials.

## 6. Conclusions

Ceramic fiber materials have the advantages of high temperature resistance, oxidation resistance, superior chemical stability, and good mechanical vibration resistance, and have been applied in aerospace, energy, metallurgy, construction, personal protection, and other thermal protection fields. In recent years, breakthroughs have been made in the refinement of ceramic fibers, flexible designs, and the development of body-shaped ceramic materials, which have simultaneously improved the lightness and thermal insulation properties of ceramic fiber materials. The research and development of these ceramic micro-nanofibrous materials could have a positive impact on the technological innovation and industrial upgrading in the field of high-temperature insulation in the future. This review starts by elaborating the thermal insulation mechanism of ceramic fiber, and then summarizes the theoretical thermal conductivity models due to solid and gaseous conductions. Subsequently, the ceramic micro-nanofibrous thermal insulation materials, which are available in a variety of dimensions, including 2D ceramic fibrous membranes and 3D ceramic fibrous aerogels, are presented in a comprehensive manner. The 2D ceramic micro-nanofiber membranes used for high-temperature insulation applications are categorized into three types: oxide ceramic fibers (e.g., SiO_2_, Al_2_O_3_, ZrO_2_, Y_2_O_3_, MgO, and others), nitride ceramic fibers (e.g., Si_3_N_4_, SiBN, SiBCN), and carbide ceramic fibers (e.g., SiC, ZrC). The construction strategies for 3D ceramic micro-nanofiber aerogels are summarized, including the direct spinning method, template method, sol-gel impregnation method, vacuum filtration molding method, freeze-drying method, and stacking method. Furthermore, this review provides a comprehensive overview of the challenges and future trends associated with ceramic micro-nanofiber materials. It is anticipated that this review could provide some valuable insights for the future development of ceramic fiber thermal insulation materials.

## Figures and Tables

**Table 1:** Theoretical thermal conductivity models due to solid and gas conductions.

Model	Structure Schematic	Equations	Ref.
Parallel model	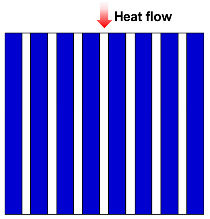	$\lambda_{c}=\omega \cdot \lambda_{s}+(1-\omega )\cdot \lambda_{g}\;$	[43,47,48,49,50,51,52]
		where λ_c_ is thermal conductivity due to solid and gas conductions, λ_g_ is the gas thermal conductivity, λ_s_ is the solid thermal conductivity, and ω is the volume fraction of solid.	
Series model	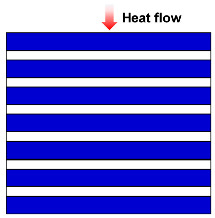	$\lambda_{c}=\frac{\lambda_{s}\cdot \lambda_{g}}{\omega \cdot \lambda_{g}+(1-\omega )\cdot \lambda_{s}}\;$	[47,53,54,55]
		where λ_c_ is thermal conductivity due to solid and gas conductions, λ_g_ is the gas thermal conductivity, λ_s_ is the solid thermal conductivity, and ω is the volume fraction of solid.	
Singh model	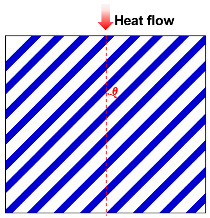	${\lambda_{c}}^{2}={\left[\omega \cdot \lambda_{s}+(1-\omega )\cdot \lambda_{g}\right]}^{2}\cdot \cos^{2}\theta +{\left[\frac{\lambda_{s}\cdot \lambda_{g}}{\omega \cdot \lambda_{g}+(1-\omega )\cdot \lambda_{s}}\right]}^{2}\cdot \sin^{2}\theta$	[56,57]
		where λ_c_ is thermal conductivity due to solid and gas conductions, λ_g_ is the gas thermal conductivity, λ_s_ is the solid thermal conductivity, ω is the volume fraction of solid, and θ is the angle between fiber orientation and heat flow direction.	
Bankvall model	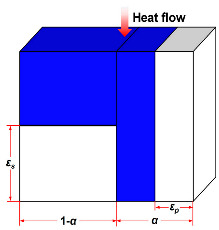	$\lambda_{c}=\alpha \cdot [\varepsilon_{p}\cdot \lambda_{g}+(1-\varepsilon_{p})\cdot \lambda_{s}]+(1-\alpha )\cdot \frac{\lambda_{s}\cdot \lambda_{g}}{\left[\varepsilon_{s}\cdot \lambda_{s}+(1-\varepsilon_{s})\cdot \lambda_{g}\right]}$	[54,58,59,60]
		where λ_c_ is thermal conductivity due to solid and gas conductions, λ_g_ is the gas thermal conductivity, λ_s_ is the solid thermal conductivity, α is the proportion of fibers parallel to heat flow, and ε_p_ and ε_s_ represent structural parameters, relating to parallel and series heat transfer mechanisms, respectively. The total porosity (ε) of the fibrous materials could be expressed as ε = α·ε_p_ + (1 − α)·ε_s_.	
Maxwell–Eucken model	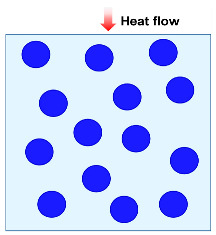	$\lambda_{c}=\frac{\lambda_{g}\cdot (1-\omega )+\lambda_{s}\cdot \omega \cdot \frac{3\lambda_{g}}{2\lambda_{g}+\lambda_{s}}}{(1-\omega )+\omega \cdot \frac{3\lambda_{g}}{2\lambda_{g}+\lambda_{s}}}$	[55]
		where λ_c_ is thermal conductivity due to solid and gas conductions, λ_g_ is the gas thermal conductivity, λ_s_ is the solid thermal conductivity, and ω is the volume fraction of solid.	
EMT model	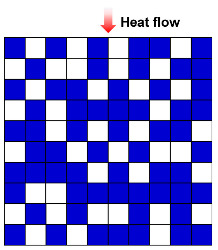	$\omega \cdot \frac{\lambda_{s}-\lambda_{c}}{\lambda_{s}+2\lambda_{c}}+(1-\omega )\cdot \frac{\lambda_{g}-\lambda_{c}}{\lambda_{g}+2\lambda_{c}}=0$	[51,61,62]
		where λ_c_ is thermal conductivity due to solid and gas conductions, λ_g_ is the gas thermal conductivity, λ_s_ is the solid thermal conductivity, and ω is the volume fraction of solid.	
Hamilton model	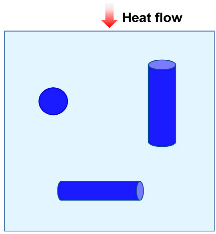	$\lambda_{c}=\lambda_{g}\cdot \frac{\lambda_{s}+(n-1)\cdot \lambda_{g}-\omega \cdot (n-1)\cdot (\lambda_{g}-\lambda_{s})}{\lambda_{s}+(n-1)\cdot \lambda_{g}+\omega \cdot (\lambda_{g}-\lambda_{s})}$	[53,61,63]
		where λ_c_ is thermal conductivity due to solid and gas conductions, λ_g_ is the gas thermal conductivity, λ_s_ is the solid thermal conductivity, and ω is the volume fraction of solid. n is an empirical shape factor. For spherical particles, n = 3; for infinitely long cylinders, n = 6.	
Bhattacharyya model	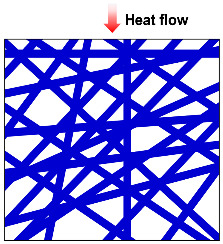	$\lambda_{c}=\lambda_{s}+\frac{\lambda_{g}-\lambda_{s}}{1+\frac{\omega }{1-\omega }\cdot \left[1+\frac{2(\lambda_{g}-\lambda_{s})}{3(\lambda_{g}+\lambda_{s})}\right]}$	[43,64,65]
		where λ_c_ is thermal conductivity due to solid and gas conductions, λ_g_ is the gas thermal conductivity, λ_s_ is the solid thermal conductivity, and ω is the volume fraction of solid.	

**Table 2:** Preparation methods and related properties of 3D ceramic fiber aerogels.

Category	Preparation Method	Calcination Parameters	Diameter (μm)	Density (mg·cm^−3^)	Porosity (%)	Thermal Conductivity Test Method	Thermal Conductivity (W·m^−1^·K^−1^)	Ref.
SiO_2_	Direct spinning method	900 °C, 60 min, Air	0.343	–	>96	Hot Disk instrument	0.0389 (25 °C)	[104]
	Vacuum filtration molding method	–	~3	80–220	90–96.4	Hot Disk method	0.023–0.037 (room temperature)	[105]
	Sol-gel impregnation method	–	8–12	226–271	87.2–91.3	Transient hot-wire method	0.0179–0.0283 (25 °C)	[106]
	Freeze-drying method	900 °C, 30 min, Air	0.206	>0.15	99.993	Hot Disk instrument	0.025–0.032 (room temperature)	[34]
Al_2_O_3_	Direct spinning method	900 °C, 2 h; 1200 °C, 2 h, Air	–	2	>99.9	Hot Disk instrument	0.022 (room temperature)	[107]
	Sol-gel impregnation method	1200 °C, 1 h, Air	0.15	146	96	25 °C: Heat flow method 200–1200: Heat flow meter apparatus	0.026 (25 °C); 0.089 (1200 °C)	[31]
ZrO_2_	Direct spinning method	800 °C, 200 min, Air	–	–	–	–	0.027 (room temperature)	[28]
TiO_2_	Sol-gel impregnation method	600 °C, 6 h, Air	0.01–0.02	304	–	Transient hot-wire technique	0.071 (1100 °C)	[108]
Mullite	Freeze-drying method	1400 °C, 2 h, Air	0.73	49.13–82.21	97.07–98.25	Hot Disk instrument	0.038–0.058 (room temperature)	[109]
	Vacuum filtration molding method	1400 °C, 1 h, Air	6	410–650	79.4–87.3	Heat flow test method	0.095 (room temperature)	[110]
	Sol-gel impregnation method	800 °C, 24 h, Air	4	360	–	Water flow plate method	0.082 (1200 °C)	[111]
	Direct spinning method	1000–1500 °C, 1 h, Air	2	15	–	Transient plane source method	0.0265 (40 °C)	[112]
	Direct spinning method	1100–1500 °C, 1 h, Air	0.29 ± 0.03	1.5	99.95	Hot Disk instrument	0.0228 (room temperature)	[27]
ZrO_2_-SiO_2_	Stacking method	1000 °C, Air	0.5	23	99.58	Hot Disk instrument	0.024 (room temperature)	[113]
	Stacking method	1000 °C, 2 h, Air	0.4–0.8	–	–	Hot Disk instrument	0.0268 (25 °C); 0.11 (900 °C)	[35]
ZrO_2_-Al_2_O_3_	Stacking method	800 °C, 2 h, Air	0.38–0.74	–	>98	Hot Disk instrument	0.0322 (room temperature)	[114]
Si_3_N_4_	Stacking method	Partial-hot-pressing at 1200 °C, 30 min, Air	0.5–1.6	1.8–9.6	~99.94	Transient hot-wire method	0.029 (room temperature)	[115]
	Freeze-drying method	–	~0.05	10	>99	Transient hot-wire method	0.0157 (room temperature)	[116]
	Template method	1300 °C, 2 h, N_2_; 700 °C, 4–7 h, Air	~0.08	8.9	96.6674	Hot Disk instrument	0.03–0.11 (room temperature)	[29]
BN	Freeze-drying method	1200 °C, 3 h, NH_3_	0.6–1.8	15.5	–	–	0.0346 ± 0.0015	[117]
	Template method	90 °C, 1 h, 500 °C, 1 h, 1500 °C, 3 h, Air	–	0.1	99.99	Homemade steady-state device	0.02	[30]
SiC	Vacuum filtration molding method	700 °C, 3 h, Air	0.03–0.28	3–19	>99	Hot Disk instrument	0.025 (room temperature)	[118]
	Template method	800 °C, 10 h, Air	0.3	26–206	84.34–98.01	Laser thermal conductivity meter	0.304 (1200 °C)	[119]
	Freeze-drying method	1000 °C, 30 min, Air	0.02–0.05	6.5	98	Laser flash apparatus	0.014	[33]
	Freeze-drying method	–	0.2	10	–	Hot Disk instrument	0.053 (50 °C)	[120]

## Data Availability

Not applicable.
